# The potential of a combination of pungent spices as a novel supplement in gilthead seabream (*Sparus aurata*) diets to aid in the strategic use of fish oil in aquafeeds: a holistic perspective

**DOI:** 10.3389/fimmu.2023.1222173

**Published:** 2023-09-25

**Authors:** Alberto Ruiz, Ignasi Sanahuja, Karl B. Andree, Dolors Furones, Paul G. Holhorea, Josep A. Calduch-Giner, Jose J. Pastor, Marc Viñas, Jaume Pérez-Sánchez, Sofia Morais, Enric Gisbert

**Affiliations:** ^1^ Aquaculture Program, Institut de Recerca i Tecnologia Agroalimentàries (IRTA), Centre de La Ràpita, La Ràpita, Spain; ^2^ Ph.D. Program in Aquaculture, Universitat de Barcelona, Barcelona, Spain; ^3^ Nutrigenomics and Fish Growth Endocrinology Group, Institute of Aquaculture Torre de la Sal, Consejo Superior de Investigaciones Científicas (CSIC), Castellón, Spain; ^4^ Innovation Division, Animal Science Unit, Lucta S.A. Bellaterra, Spain; ^5^ Sustainability in Biosystems, Institut de Recerca i Tecnologia Agroalimentàries (IRTA) Torre Marimon, Caldes de Montbui, Barcelona, Spain

**Keywords:** phytogenics, spices, anti-inflammatory, DHA/EPA ratio, digestion, bile salts, docosahexaenoic acid, microbiota

## Abstract

This work studied the potential of a combination of pungent spices (capsicum, black pepper, ginger, and cinnamaldehyde) to be used as a supplement in diets of gilthead seabream (*Sparus aurata*; 44.1 ± 4.2 g). During 90 days, fish were fed three experimental diets with low inclusion of fish oil and containing poultry fat as the main source of lipids, supplemented with graded levels of the tested supplement: 0 (control), 0.1 (SPICY_0.1%_), and 0.15% (SPICY_0.15%_). As a result, the pungent spices enhanced the growth performance, the activity of the bile-salt-activated lipase in the intestine, and decreased fat deposit levels within enterocytes. The SPICY_0.1%_ diet reduced the feed conversion ratio and the perivisceral fat index and lipid deposits in the liver. Moreover, the ratio of docosahexaenoic acid/eicosapentaenoic acid in fillet increased in fish fed the SPICY_0.1%_ diet, while the hepatic levels of docosahexaenoic acid and total n-3 polyunsaturated fatty acids increased in fish fed the SPICY_0.15%_ diet. Furthermore, there was an effect on the expression of some biomarkers related to lipid metabolism in 2-h postprandial fish (*fasn*, *elovl6*, *scd1b*, *cyp7a1*, *lpl*, and *pparβ*), and in 48 h fasted-fish fed with the SPICY_0.1%_ diet, a regulation of the intestinal immune response was indicated. However, no significant differences were found in lipid apparent digestibility and proximate macronutrient composition. The spices did not affect biomarkers of hepatic or oxidative stress. No differences in microbial diversity were found, except for an increase in Simpson’s Index in the posterior intestine of fish fed the SPICY_0.1%_ diet, reflected in the increased relative abundance of the phylum Chloroflexi and lower relative abundances of the genera *Campylobacter*, *Corynebacterium*, and *Peptoniphilus*. In conclusion, the supplementation of gilthead seabream diets with pungent spices at an inclusion of 0.1% was beneficial to enhance growth performance and feed utilization; reduce fat accumulation in the visceral cavity, liver, and intestine; and improve the fish health status and condition. Results suggest that the tested supplement can be used as part of a nutritional strategy to promote a more judicious use of fish oil in fish diets due to its decreasing availability and rising costs.

## 1 Introduction

It is well-established that n-3 long-chain polyunsaturated fatty acids (n-3 LC-PUFAs), particularly eicosapentaenoic (C20:5n-3; EPA) and docosahexaenoic (C22:6 n-3; DHA) acids, have multiple beneficial effects in vertebrate health, such as promoting a correct neurophysiological development and in the prevention of cancers, cardiovascular, and inflammatory diseases (1–3). The main source for humans of these n-3 LC-PUFAs has traditionally been the dietary intake of fish and seafood. However, fish are also dependent on their dietary intake, since many marine fishes have a restricted ability to biosynthesize DHA and EPA due to their low capacity to desaturate and elongate linoleic (C18:2 n-6) and alpha-linolenic (C18:3 n-3) acids (4). Thus, n-3 LC-PUFAs have been usually incorporated to aquafeeds as fish oil. Nevertheless, the decreasing availability of fish oil to sustain the growth of aquaculture worldwide, coupled with its rising cost, have been a strong driver to search for functional ingredients to reduce fish oil in aquafeeds while maintaining fish health and welfare, and n-3 LC-PUFA levels within the recommended and expected range for the consumer.

During recent decades, the use of plant-based oils as an alternative source to fish oils for dietary energy has been widespread among aquafeed manufactures, given their lower cost and higher availability. The partial replacement of fish oil by vegetal oils does not usually affect fish performance in terms of growth and feed utilization (5, 6). Nonetheless, some studies have reported important changes in the fatty acid profile of the fillet, with a reduction in the levels of EPA and DHA and an increase in alpha-linolenic, linoleic, and oleic (C18:1 n-9) acids (5–7). Rendered fats from animals are another affordable and widely available resource, with a growing interest in its improved use in the context of a circular bioeconomy. Moreover, besides containing more n-3 LC-PUFAs and less n-6 PUFAs than plant-based oils (8), they have a higher level of saturated fatty acids (SFAs) and monounsaturated fatty acids (MUFAs), which are preferential substrates for metabolic energy production (β-oxidation), sparing LC-PUFAs from catabolism (9). For these reasons, rendered animal fats have been advocated as a better alternative, compared to vegetable oils, to partially replace fish oil in fish feeds. Nonetheless, the substitution of fish oil by rendered animal fats still leads to a reduction in the levels of DHA and EPA in the fillet, even if less marked than with vegetable oils, although it does not normally compromise fish performance (10). Furthermore, n-3 LC-PUFA are important regulators of metabolic pathways, with well-known hypolipidemic and hypocholesteremic effects in vertebrates (11), including fish (12). Therefore, fish oil replacement by either vegetal or land-based animal oils can cause important changes in lipid and energy metabolism (13, 14), which may lead to physiological disorders, such as a high accumulation of fat deposits within enterocytes, hepatic vacuolization and steatosis, and high levels of perivisceral fat (10, 15).

However, the reduction in fish oil levels in aquafeeds is inevitable, and although alternative sources of n-3 LC-PUFA are emerging (e.g., algal oils, heterotrophic single cell organisms, genetically modified oilseeds), available volumes and production costs are still not at a level that they could bridge the gap in the near future. Therefore, in the meantime, new strategies are required to mitigate some of the negative effects associated with the reduction in fish oils in fish diets. The present study explores the use of supplements with a lipotropic function. Several studies have demonstrated that the incorporation of ingredients with a lipotropic function in fish diets can regulate the fishes’ lipid profile, reducing physiological disorders such as inflammation and improving their performance (16, 17). Considering studies in higher vertebrates, spices are promising candidates as feed additives, which should be further explored in this context. Pungent vegetal substances are valued in cooking for their intense flavor and aroma and for their properties as food preservatives, but they also have many well-demonstrated physiological benefits (18, 19). For instance, mammalian studies have established that pungent spices act as hypocholesterolemic and hypotriglyceridemic agents (similarly to n-3 LC-PUFA in fish oils) and stimulate digestion, and having antioxidant, antimicrobial, and anti-inflammatory effects. Thanks to these properties, spices are also commonly used as nutraceuticals in humans, with many therapeutic and prophylactic applications, such as prevention or reduction in obesity, diabetes, and carcinogenesis, among others (18, 19).

The aim of this work is to offer a holistic insight of the effects of supplementing a reduced fish oil diet containing poultry fat as the major lipid source (high in SFAs) with a product based on a combination of capsicum, black pepper, and ginger oleoresins, and cinnamaldehyde, in terms of fish performance and overall physiological status, including nutrient metabolism, immune response, and gut microbial profile. The study was performed in gilthead seabream (*Sparus aurata*), since this is one of the most important farmed marine fish species in the Mediterranean area.

## 2 Materials and methods

### 2.1 Ethics statement

All procedures involving fish manipulation and tissue sampling complied with the Spanish (law 32/2007 and Royal Decree 1201/2015) and the current European legislation (EU2010/63) and were authorized by the Ethical Committee of the Institute of Agrifood Research and Technology and the Generalitat of Catalunya (CEEA 219/2020).

### 2.2 Animals, diets, and experimental design

The 90-day feeding trial was carried out at the Institute of Agrifood Research and Technology (IRTA) in La Ràpita (Tarragona, Spain). After an acclimation period of 2 weeks, juveniles of gilthead seabream (initial body weight, BW_i_ = 44.1 ± 4.2 g; mean ± standard deviation, SD) obtained from a commercial fish farm (Piscicultura Marina Mediterranea SL, Andromeda Group, Valencia, Spain) were randomly distributed in 12 tanks of 450 L (30 fish per tank; initial density = 3 kg m^−3^; N = 360). To guarantee water quality maintenance, tanks were connected to an IRTAmar™ water recirculation system, following the natural photoperiod (June to August at 40.63N–0.66E). Water temperature, dissolved oxygen, and pH were kept at 22.5 ± 0.5°C, 6.3 ± 0.2 mg/L (OXI330, Crison Instruments, Barcelona, Spain) and 7.6 ± 0.01 (pH meter 507, Crison Instruments), respectively. Salinity, nitrite, and ammonia levels were 36‰ (MASTER-20 T Hand-Held Refractometer, ATAGO Co. Ltd., Italy), 0.16 ± 0.1 mg NO_2_^−^/L, and 0.22 ± 0.08 mg NH_4_^+^/L (HACH DR 900 Colorimeter, Hach Company, Spain), respectively.

Three experimental diets (**Table 1**; 3 mm pellet size) were designed to evaluate the potential use of an encapsulated combination of capsicum, black pepper, and ginger oleoresins, and cinnamaldehyde (Lucta S.A., Spain; Patent Number WO/2022/117810) as a feed additive. The encapsulation serves to enable the manipulation of the pungent spices during feed preparation but has no functional effect (in the animal), as the active ingredients should be released during the extrusion process. A basal diet was formulated, which included a high content of poultry fat and lower levels of fish oil and soybean oil. This formula was chosen to increase the levels of SFAs (27% of total fatty acids; **Table 1**) compared to traditional fish oil replacement strategies based on vegetable oils, and was hypothesized to result in increased body fat deposition. The two other experimental diets had the same ingredient formulation and proximate composition, but were supplemented with the encapsulated product at two different inclusion levels before extrusion: 0.1 (SPICY_0.1%_) and 0.15% (SPICY_0.15%_). The choice of these inclusion levels was based on a previous dose–response trial performed in the same species in which the same combination of pungent spices was tested at doses between 0.05% and 0.15% (20), obtaining the best results in performance at 0.1% and 0.15%. In particular, at these inclusion levels, an improvement on feeding efficiency and lipid apparent digestibility, a reduction in hepatic lipid stores, decreased fat accumulation in fillet, and increased levels of n-3 PUFA (including EPA and DHA) in fillet. The three diets were isonitrogenous (44% crude protein), isolipidic (18% crude fat), and isoenergetic (21.4 MJ/kg), and were manufactured by Sparos Lda. (Portugal) following the procedures described by Salomón et al. (21). Yttrium oxide (Y_2_O_3_, Sigma Aldrich, Spain) was included in the diets at 0.2 g/kg as an inert marker to assess apparent digestibility coefficients (ADCs) of macronutrients. Each experimental diet was randomly assigned to four tanks before the beginning of the nutritional assay. During the 90-day trial, feed was distributed twice a day in 12 takes spread over an hour (one each 5 min) with automatic feeders (Arvo-Tec T Drum 2000, Finland).

**Table 1:** Ingredient formulation, proximate, and fatty acid composition of experimental diets: a control and two basal diets supplemented with a mixture of pungent spices (capsicum, black pepper, ginger, and cinnamaldehyde) at a dietary inclusion level of 0.1 (SPICY_0.1%_) and 0.15% (SPICY_0.15%_).

	Experimental diets		
Ingredients (%)	Control	SPICY_0.1%_	SPICY_0.15%_
Fishmeal Super Prime	7.50	7.50	7.50
Fishmeal 60	5.00	5.00	5.00
Fish protein concentrate	2.00	2.00	2.00
Feathermeal hydrolysate	5.00	5.00	5.00
Porcine blood meal	3.00	3.00	3.00
Poultry meal	15.00	15.00	15.00
Aminopro NT70—*C. glutamicum*	4.00	4.00	4.00
Corn gluten meal	8.00	8.00	8.00
Soybean meal 48	12.00	12.00	12.00
Sunflower meal	5.00	5.00	5.00
Wheat meal	10.31	10.31	10.31
Whole peas	5.00	5.00	5.00
Pea starch (raw)	2.40	2.40	2.40
Fish oil	3.02	3.02	3.02
Soybean oil	2.35	2.35	2.35
Poultry fat	8.04	8.04	8.04
Vitamin and mineral premix	1.00	1.00	1.00
Vitamin C35	0.05	0.05	0.05
Vitamin E50	0.02	0.02	0.02
Betaine HCl	0.20	0.20	0.20
Choline chloride 60	0.10	0.10	0.10
Antioxidant	0.20	0.20	0.20
Sodium propionate	0.10	0.10	0.10
Monoammonium phosphate	0.35	0.35	0.35
L-Tryptophan	0.15	0.15	0.15
DL-Methionine	0.20	0.20	0.20
Mixture of pungent spices (Lucta)	-	0.10	0.15
Yttrium oxide	0.02	0.02	0.02
Proximate composition			
Crude protein, %	44.14 ± 0.05	44.11 ± 0.06	44.26 ± 0.21
Crude fat, %	18.10 ± 0.04	18.09 ± 0.06	18.19 ± 0.09
Gross energy, MJ kg^−1^	21.38 ± 1.11	21.46 ± 0.92	21.45 ± 0.83
Fatty acid profile (% of total fatty acids) *			
Saturated fatty acids (SFAs)	27.19 ± 0.40	27.79 ± 0.01	27.76 ± 1.36
Monounsaturated fatty acids (MUFAs)	36.61 ± 0.73	35.97 ± 0.59	36.18 ± 0.51
n-6 polyunsaturated fatty acids (n-6 PUFAs)	26.65 ± 0.06	26.69 ± 0.45	26.45 ± 0.52
n-3 polyunsaturated fatty acids (n-3 PUFAs)	9.55 ± 0.39	9.55 ± 0.03	9.61 ± 0.00
Total PUFAs	36.20 ± 0.45	36.24 ± 0.48	36.06 ± 0.52

*Complete fatty acid profile of experimental diets is detailed in **Supplementary Table S1**. The proximate and fatty acid composition of diets were analyzed in duplicate; values are represented as mean ± standard deviation (SD).

Once a month, fish were anesthetized with buffered tricaine methanesulfonate (MS-222, Sigma-Aldrich, Spain; 100 mg/L) for measuring growth in body weight (BW) and standard length (SL) in order to monitor somatic growth. In addition, the uneaten dried pellets from each tank were collected and weighed to ensure that sufficient amount of feed was being offered and to calculate the daily feed intake (22). For assessing ADCs of lipids and proteins, feces were collected by means of a sedimentation column 10–12 h after removal of uneaten feed during three consecutive days. Then, pooled samples from the same tank were frozen at −20°C until biochemical analyses.

### 2.3 Sampling and fish performance indicators

At the end of the trial, fish were fasted for 48 h and anesthetized with 100 mg/L of MS-222, and their final body weight (BW_f_) and standard length (SL_f_) were individually measured. The following key performance indicators were also calculated:


$$\text{Specific growth rate }\left(\text{SGR};\text{ \%}/\text{day}\right)\;=\;100\times \text{ln }\left[\text{B}{\text{W}}_{\text{f}}\left(\text{g}\right)\;-\text{ ln B}{\text{W}}_{\text{i}}\left(\text{g}\right)\right]/\text{time }\left(\text{days}\right).$$

Fulton's condition factor (K) = 100×BWf(g)/SLf(g)3

Feed intake (FI, g/fish) = total feed intake per tank (g)/number of fish per tank (g)

Feed conversion ratio (FCR) = total feed intake per tank (g)/fish biomass increase per tank (g)

In addition, eight fish per tank (32 per dietary treatment) were randomly selected and euthanized with an overdose of MS-222 (300 mg/L) for sampling different tissues for the analyses described below. In particular, the perivisceral fat was gently separated from the gastrointestinal tract in six fish per tank (four replicate tanks per diet) with the aid of a round-ended scalpel and individually weighed to calculate the perivisceral fat index (PVFI; %) = perivisceral fat weight (g)/BW_f_ (g). The livers of six fish per tank were also weighed to calculate the hepatosomatic index (HSI; %) = liver weight (g)/BW_f_ (g). Then, a piece of liver (1.5–2 cm^2^) and a piece of anterior intestine (AI; approximately 4 cm) from three individuals per tank (12 fish per diet) were fixed in 10% neutral buffered formalin (pH = 7.2) and stored at 4°C until histological analysis. The AI was selected because this region of the intestine has high rates of fat digestion and absorption (23). The rest of the liver was divided in pieces and frozen at −80°C to further assess hepatic antioxidant status and metabolic biomarkers (four replicate tanks per diet). The fillet and the liver of three fish per tank were stored at −20°C until analyses of proximate and fatty acid composition. To evaluate the profile of bile acids (BAs), the walls of the gallbladders of four fish per tank were broken with the edge of a scalpel, and the content of all fish from the same tank were emptied together into one tube (four replicate tanks per diet) and frozen at −80°C. With the purpose of studying the effect of the diet on hepatic and intestinal gene expression profile of 48 h fasted fish, a piece of the liver and a piece of AI (approximately 1 and 2 cm^2^, respectively) of two individuals per tank (eight per dietary treatment) were separately immersed in 5 volumes of RNAlater^®^ (Sigma-Aldrich, USA), incubated at 4°C for 24 h and stored at −80°C. A section of AI (approximately 4 cm long cut from the pyloric caeca) and posterior intestine (PI; approximately 4 cm long cut from the anus anteriorly) from three fish per tank (12 per treatment) were taken and opened lengthwise under sterile conditions. The mucosal content was gently scraped with a round-edge spatula and immediately frozen at −80°C for further microbial analysis. Sampling was performed at 48 h after the last feeding to ensure sample stability and avoid contamination by allochthonous bacteria from the feces in the case of intestinal microbiota (24).

The rest of the fish were returned to their respective tanks and fed for 3 days. Then, 10 2-h postprandial fish per tank (40 per treatment) were netted and euthanized with 300 mg/L of MS-222 for tissue sampling. The luminal content of the AI of four fish per tank was stripped with tweezers into one tube (four replicates per diet) and frozen at −80°C for future analysis of the BA profile. The digestive tract of four fish per tank was divided in two regions: i) the stomach and pyloric caeca and ii) the AI. Both regions were separately stored at −80°C until analysis of pancreatic digestive enzymes. A piece of the liver and AI from two fish per tank (eight per treatment) were dissected and conserved as previously described for gene expression analysis.

### 2.4 Proximate and fatty acid composition

For biochemical analysis, a pool of three liver pieces per tank were homogenized together, while three fillets per tank were individually homogenized (IKA T25 digital ULTRA-TURRAX, IKA Works, USA). The protocols described by Lowry et al. (25), Folch et al. (26), Dubois et al. (27), and AOAC (28) were followed to determine the levels of total protein, lipids, carbohydrates, and ash content in livers and fillets. Fatty acid profile was obtained as described by Ramos-Júdez et al. (29). In brief, transmethylated fatty acids from total lipids were extracted, purified, and finally quantified by gas-liquid chromatography on a Thermo Trace GC (Thermo Fisher, Spain) coupled to a TRACE™ TR-FAME GC Column (Thermo Scientific, Spain), using heneicosylic acid (21:0) as internal standard (ref. H5,149, Sigma-Aldrich, Spain).

Protein and lipid ADCs were calculated according to Cheng and Hardy (30) using Y_2_O_3_ as inert marker:


$$\begin{array}{l} \text{ADC of nutrient }\left(\%\right)\;=\;100\;\times \;\left[1\;-\;\left(\text{\% }{\text{Y}}_{2}{\text{O}}_{3}\text{ in diet}/\text{\% }{\text{Y}}_{2}{\text{O}}_{3}\text{ in feces}\right)\;\times \;\left(\text{\% nutrient in feces}/\text{\% nutrient in diet}\right)\right] \end{array}$$

The Y_2_O_3_ concentration was determined using an Agilent 7700 ICP-MS (Agilent Technologies, USA).

### 2.5 Hepatic metabolism and antioxidant stress

Biomarkers of hepatic metabolic and antioxidant stress were evaluated following the methodology described by Ruiz et al. (31). In brief, homogenized pools of three liver pieces (approximately 100 mg each) per tank resuspended in a lysis solution (1.24 mM Triton X-100, 1 mM EDTA, and 1 mM NaHCO_3_) with a stabilizer solution (3.7 mM EDTA, 5 mM β-mercaptoethanol), 1:1 *v/v*, were centrifuged (5,000×*g*, 10 min, 4°C), and supernatants were collected. Lactate dehydrogenase, aspartate transaminase, and alanine transaminase activities were quantified following the methodology of Bergmeyer and Bernt (32–34) with commercial kits (ref. 41,222, ref. 41,272, ref. 41,282; Spinreact, Spain). To evaluate the antioxidant condition of the liver, homogenized pools of three pieces (approximately 60 mg each) per tank resuspended in 5 volumes *v/w* of buffer (150 mM KCl, 1 mM EDTA, pH 7.4) were centrifuged (9,000×*g*, 30 min, 4°C), and the supernatants were collected. Then, superoxide dismutase (SOD), catalase (CAT), and glutathione reductase (GR) activities were quantified following the protocols of McCord and Fridovich (35), Aebi (36), and Carlberg and Mannervik (37). Lipid peroxidation (LPO) levels were estimated through thiobarbituric acid reactive substances (TBARs) as described by Solé et al. (38), and total antioxidant capacity (TAC) was measured following the manufacturer’s instructions of the Total Antioxidant Capacity Assay Kit (ref. MAK187, Sigma-Aldrich, USA). The above-mentioned oxidative stress biomarkers were normalized to soluble protein content (39), except for SOD activity, which was expressed as percent of enzyme inhibition. All described measures were run in triplicate at 25°C by UV/Vis spectrophotometry (Infinite M200 Plate Reader, Tecan Switzerland) and analyzed with the Magellan™ software (v6, Tecan).

### 2.6 Pancreatic digestive enzymes

Pools of four samples of i) stomach and pyloric ceca and ii) AI were separately homogenized (IKA T25 digital ULTRA-TURRAX, IKA Works) in 5 volumes *v/w* of ice-cold distilled water and centrifuged (3,300×*g*, 3 min, 4°C), and the supernatant was collected for quantification of pancreatic digestive enzymes (total alkaline proteases, α-amylase, and bile-salt-activated lipase) following the guidelines of Ruiz et al. (31). Samples (enzymatic crude extracts) were handled according to Solovyev and Gisbert (40) in order to prevent their degradation during storage and handling.

### 2.7 Bile acid quantification

Bile acid (BA) quantification was performed as previously described by Herrero-Encinas et al. (41) with modifications. BA extraction from gallbladder bile samples was performed by extraction of 100 µL of water-diluted bile (1/2,000) with 400 µL of acetonitrile (ACN) containing internal standard (chenodeoxycholic acid-d4, CDCA-d4). After vortex and centrifugation, supernatants were diluted 1/10 in H_2_O:ACN (1:1 *v/v*) and directly injected in the liquid chromatograph–mass spectrometer (LC-MS). Intestinal digesta samples were first lyophilized and homogenized on a TissueLyzer II (QIAGEN, Germany). Then, 20 mg of homogenate was extracted with 800 μL of H_2_O:ACN (1:1 *v/v*) including the internal standard for 15 min and centrifuged (15,000×*g*, 10 min, 4°C), and the supernatants diluted again in H_2_O:ACN (1/1,000). Quantification of BAs was performed by LC-MS using response comparison against calibration curves generated using pure BA standards. Chromatographic separation was performed on an ACQUITY UPLC I-Class connected to a Xevo-G2 QTof mass spectrometer, using QuanLynx v4.2 software for operations and quantification (Waters Corp., USA).

### 2.8 Histological analyses

To evaluate the histological condition, small segments of fixed liver and AI (approximately 0.5–1 cm^2^) were dehydrated in ethanol solutions of graded concentrations, cleared with xylene, and embedded in paraffin. Serial sections of 4 µm stained with hematoxylin and eosin were examined under light microscopy (Leica DM LB, Leica Microsystems) by means of a digital camera at 600 dpi (Olympus DP70, Olympus Europa, Germany). Inflammation and accumulation of fat deposits were semi-quantitatively evaluated from 1 to 5 following the classification described by Ruiz et al. (31). Semi-quantitative analyses were performed following a random order of samples, under blinded conditions by two different observers (42). In the images of AI, the following parameters were also measured using the software ANALYSIS (Olympus Soft Imaging Solutions, Germany): thickness of musculature, height of villi, height of enterocytes, and density of goblet cells in the intestinal mucosa (43).

### 2.9 Extraction of DNA and analysis of gut microbiota

The DNeasy PowerSoil Pro Kit (ref. 47,016, QIAGEN, Germany) was used for extracting the DNA of up to 250 mg of the scraped product of the AI and the PI of three fish from each tank (12 individuals per diet). The concentration of DNA ranged up to 500 ng/µL, and A_260_/A_280_ absorbance ratios were higher than 1.85.

The region V3–V4 of the 16S rRNA gene was amplified (primers 341F/805R) and sequenced (llumina-MiSeq platform; 2 × 300 bp paired-end) according to Ruiz et al. (44), and data analysis was carried out with a workflow based on the R package dada2 (v1.16; 45). In brief, all reads with a Phred quality score <28 or with an expected error >2 were excluded from the analysis. After merging of paired-ended reads, the sequences with an overlap length <12 nucleotides, more than 0 mismatches, or identified as chimeras, were also removed. Finally, for bacterial taxonomy classification of amplicon sequence variants (ASVs), the SILVA database (v138.1) was used as a reference library. Those ASVs with a bootstrapping confidence <80% were classified as unassigned (46). According to rarefaction curves (**Supplementary Figure S1**), the number of reads per sample were rarefied to the minimum sample depth (49,337 reads) using the R package vegan (v2.6-4) and normalized by total sum scaling (47). Raw sequencing data are available in the Sequence Read Archive (SRA) of NCBI under the Bioproject accession numbers PRJNA915342 and PRJNA971862.

### 2.10 Gene expression profile of the liver and anterior intestine

The TRI Reagent (Sigma-Aldrich, USA) and QIAGEN RNeasy^®^ Mini Kit (ref. 74,106, QIAGEN, Germany) were, respectively, used for extracting RNA from the liver and AI. Concentrations of RNA ranged between 20 and 100 ng/µL, with A_260_/A_280_ absorbance ratios of 1.9–2.1 (Nanodrop-2000^®^, Thermo Fisher Scientific, USA). Integrity was verified through agarose gel electrophoresis (48). For cDNA synthesis, the High-Capacity cDNA Archive Kit (Applied Biosystems, USA) was used following the manufacturer’s instructions with an initial input of 500 ng of RNA.

Real-time quantitative PCR was carried out with a CFX96 Connect^™^ Real-Time PCR Detection System (Bio-Rad, USA), using 96-well PCR array layouts designed for simultaneously profiling a panel of 44 genes for liver (**Table 2**) and intestine (**Table 3**) as described by Ruiz et al. (44). To improve data reproducibility, all the pipetting operations were performed using an EpMotion 5070 Liquid Handling Robot (Eppendorf, Germany). Expression values were calculated with the delta–delta Ct method (49), taking *beta-actin* as a housekeeping gene, after testing its expression stability (GeNorm software; M score = 0.21). To compare the expression of multiple genes, all values in the liver and AI were referenced to the expression levels of *grp-170* and *hes1-b* of fish fed the control diet, respectively.

**Table 2:** PCR-array layout for gene expression profile in the liver of gilthead seabream fed experimental diets.

Function	Gene	Symbol	GenBank
Fatty acids, cholesterol and phospholipid metabolism	Fatty acid synthase	*fasn*	JQ277708
	Elongation of very long chain fatty acids 1	*elovl1*	JX975700
	Elongation of very long chain fatty acids 4	*elovl4*	JX975701
	Elongation of very long chain fatty acids 5	*elovl5*	AY660879
	Elongation of very long chain fatty acids 6	*elovl6*	JX975702
	Fatty acid desaturase 2	*fads2*	AY055749
	Stearoyl-CoA desaturase 1a	*scd1a*	JQ277703
	Stearoyl-CoA desaturase 1b	*scd1b*	JQ277704
	Cholesterol 7-alpha-monooxygenase	*cyp7a1*	KX122017
	Phospholipid transfer protein	*pltp*	XM_030418561
Lipases	Adipose triglyceride lipase	*atgl*	JX975711
	Hepatic lipase	*hl*	EU254479
	Lipoprotein lipase	*lpl*	AY495672
	85kDa calcium-independent phospholipase A2	*pla2g6*	JX975708
Transcription factors & nuclear receptors	Hepatocyte nuclear factor 4 alpha	*hnf4a*	FJ360721
	Sterol regulatory element-binding proteins 1	*srebp1*	JQ277709
	Sterol regulatory element-binding protein 2	*srebp2*	XM_030408996
	Farnesoid X receptor	*fxr*	XM_030426192
	Liver X receptor α	*lxrα*	FJ502320
	Peroxisome proliferator-activated receptor α	*pparα*	AY590299
	Peroxisome proliferator-activated receptor β	*pparβ*	AY590301
	Peroxisome proliferator-activated receptor γ	*pparγ*	AY590304
Oxidative metabolism & energy sensing	Carnitine palmitoyltransferase 1A	*cpt1a*	JQ308822
	Hydroxyacyl-CoA dehydrogenase	*hadh*	JQ308829
	Fatty acid translocase/CD36	*fat/cd36*	XM_030440140
	Fatty acid binding protein, heart	*h-fabp*	JQ308834
	Citrate synthase	*cs*	JX975229
	NADH-ubiquinone oxidoreductase chain 2	*nd2*	KC217558
	NADH-ubiquinone oxidoreductase chain 5	*nd5*	KC217559
	Cytochrome c oxidase subunit I	*coxi*	KC217652
	Proliferator-activated receptor gamma coactivator 1 alpha	*pgc1α*	JX975264
	Sirtuin1	*sirt1*	KF018666
	Sirtuin2	*sirt2*	KF018667
Antioxidant defense	Catalase	*cat*	JQ308823
	Uncoupling protein 1	*ucp1*	FJ710211
	Glutathione peroxidase 1	*gpx1*	DQ524992
	Glutathione peroxidase 4	*gpx4*	AM977818
	Peroxiredoxin 3	*prdx3*	GQ252681
	Peroxiredoxin 5	*prdx5*	GQ252683
	Superoxide dismutase [Cu-Zn]	*cu-zn-sod/sod1*	JQ308832
	Superoxide dismutase [Mn]	*mn-sod/sod2*	JQ308833
	Glucose-regulated protein, 170 kDa	*grp-170*	JQ308821
	Glucose-regulated protein, 94 kDa	*grp-94*	JQ308820
	Glucose-regulated protein, 75 kDa	*grp-75*	DQ524993

Specific primer sequences for marker genes of liver are listed in **Supplementary Table S2**.

**Table 3:** PCR-array layout for gene expression profile in the intestine of gilthead seabream fed experimental diets.

Function	Gene	Symbol	GenBank
Epithelial integrity	Proliferating cell nuclear antigen	*pcna*	KF857335
	Transcription factor HES-1-B	*hes1-b*	KF857344
	Krüppel-like factor 4	*klf4*	KF857346
	Claudin-12	*cldn12*	KF861992
	Claudin-15	*cldn15*	KF861993
	Cadherin-1	*cdh1*	KF861995
	Cadherin-17	*cdh17*	KF861996
	Tight junction protein ZO-1	*tjp1*	KF861994
	Desmoplakin	*dsp*	KF861999
	Gap junction Cx32.2 protein	*cx32.2*	KF862000
	Coxsackievirus and adenovirus receptor homolog	*cxadr*	KF861998
Nutrient transport	Intestinal-type alkaline phosphatase	*alpi*	KF857309
	Liver type fatty acid-binding protein	*fabp1*	KF857311
	Intestinal fatty acid-binding protein	*fabp2*	KF857310
	Ileal fatty acid-binding protein	*fabp6*	KF857312
Mucus production	Mucin 2	*muc2*	JQ277710
	Mucin 13	*muc13*	JQ277713
Interleukins	Tumor necrosis factor-alpha	*tnf-α*	AJ413189
	Interleukin-1 beta	*il-1β*	AJ419178
	Interleukin-6	*il-6*	EU244588
	Interleukin-7	*il-7*	JX976618
	Interleukin-8	*il-8*	JX976619
	Interleukin-10	*il-10*	JX976621
	Interleukin-12 subunit beta	*il-12β*	JX976624
	Interleukin-15	*il-15*	JX976625
	Interleukin-34	*il-34*	JX976629
Cell markers	Cluster of differentiation 4-1	*cd4-1*	AM489485
	Cluster of differentiation 8 beta	*cd8b*	KX231275
	C–C chemokine receptor type 3	*ccr3*	KF857317
	C–C chemokine receptor type 9	*ccr9*	KF857318
	C–C chemokine receptor type 11	*ccr11*	KF857319
	C–C chemokine CK8/C–C motif chemokine 20	*ck8/ccl20*	GU181393
	Macrophage colony-stimulating factor 1 receptor 1	*csf1r1*	AM050293
Ig production	Immunoglobulin M	*igm*	JQ811851
	Immunoglobulin T membrane-bound form	*igt-m*	KX599201
Pattern recognition	Galectin-1	*lgals1*	KF862003
receptors (PRRs)	Galectin-8	*lgals8*	KF862004
	Toll-like receptor 2	*tlr2*	KF857323
	Toll-like receptor 5	*tlr5*	KF857324
	Toll-like receptor 9	*tlr9*	AY751797
	CD209 antigen-like protein D	*cd209d*	KF857327
	CD302 antigen	*cd302*	KF857328
	Macrophage mannose receptor 1	*mrc1*	KF857326
	Fucolectin	*fcl*	KF857331

Specific primer sequences for marker genes of intestine are listed in **Supplementary Table S3**.

### 2.11 Statistical analyses

After confirmation of normal distribution and homoscedasticity of data by Shapiro–Wilk test and Levene’s test, a one-way ANOVA followed by Tukey’s range test for multiple comparison among groups (*p* ≤ 0.05) was performed. When data was non-parametric, a Kruskal–Wallis one-way analysis of variance by ranks and Dunn’s *post-hoc* test were performed. Correlations between variables were tested by the Pearson product–moment correlation test (*p* ≤ 0.05).

For gene expression analyses, a Student’s t-test was performed. A two-way ANOVA followed by a Holm–Sidak test was used for evaluating interaction between the diet and feeding time (2 h postprandial or 48 h fasted). The *p*-value was set to 0.05 to determine significant differences among dietary groups, whereas *p* ≤ 0.1 was considered as a tendency for gene expression data. In the case of gene expression values with *p* ≤ 0.1, a partial least squares-discriminant analysis (PLS-DA) was constructed with the software EZinfo (v3.0, Umetrics, Sweden) to achieve the maximum separation among experimental groups. Cluster separation was assessed by calculating Hotelling’s T^2^ statistic. Points with a T^2^ above 95% confidence limit were considered as outliers and discarded. By means of the R package *ropls* (v1.22.0), each PLS-DA model was validated by a permutations test, making sure that there was no over-fitting (**Supplementary Figures S2, S3**).

Regarding gut microbial communities, significant differences among dietary groups in alpha diversity metrics (indices of Chao1, ACE, Shannon, Simpson, and Faith’s phylogenetic diversity; 50, 51) were determined by Wilcoxon test (*p* ≤ 0.05). As a beta diversity index, the weighted UniFrac distance was used to estimate similarities among samples based on the phylogenetic relationships of their ASVs (52). A permutational multivariate analyses of variance (PERMANOVA) was performed to check significant differences in beta diversity (*p* ≤ 0.05; 53). Differential abundances among groups in phyla and genera with a relative abundance ≥1% were calculated with the method Metastats, adjusting the *p*-value by False Discovery Rate (FDR) (54). All the described statistics for gut microbiota data were executed with the R package microeco (55), which was used together with ggplot2 for generation of figures.

## 3 Results

### 3.1 Fish performance

The supplementation of pungent spices into the basal diet had a positive effect in fish somatic growth in terms of BW_f_ and SGR at both dietary inclusion levels when compared to the control diet (**Table 4**; *p* < 0.05). Furthermore, the administration of the SPICY_0.1%_ diet reduced perivisceral fat levels (*p* < 0.05), while fish fed the SPICY_0.15%_ diet showed PVFI values that were intermediate between the abovementioned and the control diet (*p* > 0.05). No significant differences were found in the values of SL_f_, Fulton’s condition factor, and HSI among dietary groups (*p* > 0.05). Regarding feed performance, whereas there were no differences in feed intake (*p* > 0.05), FCR values followed a similar pattern to that described for the PVFI; in particular, the lowest FCR values were found in fish fed the SPICY_0.1%_ diet (*p* < 0.05). On the other hand, diet supplementation with pungent spices did not significantly affect lipid and protein ADCs (*p* > 0.05).

**Table 4:** Growth and feed performance indicators, somatic condition indices, and macronutrient apparent digestibility coefficients of gilthead seabream fed the control and two basal diets supplemented with a mixture of pungent spices (capsicum, black pepper, ginger, and cinnamaldehyde) at a dietary inclusion level of 0.1 (SPICY_0.1%_) and 0.15% (SPICY_0.15%_).

	Control	SPICY_0.1%_	SPICY_0.15%_
Growth performance			
BW_i_ (g)	44.05 ± 0.04	44.08 ± 0.10	44.06 ± 0.10
SL_i_ (cm)	12.04 ± 0.12	12.22 ± 0.16	12.08 ± 0.06
BW_f_ (g)	215.80 ± 1.06^a^	221.96 ± 3.46^b^	223.02 ± 3.50^b^
SL_f_ (cm)	19.32 ± 0.21	19.66 ± 0.30	19.51 ± 0.09
SGR (% day^−1^)	1.81 ± 0.01^a^	1.84 ± 0.02^b^	1.84 ± 0.02^b^
Somatic indices			
K	3.00 ± 0.11	2.93 ± 0.12	3.01 ± 0.07
HSI (%)	1.84 ± 0.11	1.92 ± 0.09	1.95 ± 0.14
PVFI (%)	3.01 ± 0.28^b^	2.32 ± 0.37^a^	2.68 ± 0.28^ab^
Feed performance			
FI (g fish^−1^)	195.89 ± 8.70	192.29 ± 9.94	198.50 ± 6.23
FCR	1.21 ± 0.05^b^	1.13 ± 0.02^a^	1.20 ± 0.04^ab^
Apparent digestibility coefficients (ADCs)			
Lipid ADC (%)	81.60 ± 0.96	81.14 ± 1.33	79.73 ± 0.89
Protein ADC (%)	79.41 ± 3.60	81.09 ± 2.82	76.08 ± 2.18

Values are represented as mean ± SD (n = 4 tanks per dietary group) and differences among groups (p ≤ 0.05) are indicated by the different superscript letters. BW_i_, initial body weight; SL_i_, initial standard length; BW_f_, final body weight; SL_f_, final standard length; SGR, specific growth rate; K, Fulton’s condition factor; HSI, hepatosomatic index; PVFI, perivisceral fat index; FI, feed intake; FCR, feed conversion ratio.

### 3.2 Proximate and fatty acid composition

There were no differences in the proximate composition of the liver nor fillets among dietary groups (**Supplementary Table S4**; *p* > 0.05). Considering the fatty acid profile of the liver, only DHA was found at different levels among dietary groups. In particular, the highest levels of DHA were found in fish fed the SPICY_0.15%_ diet (*p* < 0.05), while fish fed the SPICY_0.1%_ diet displayed intermediate values between both dietary treatments (**Table 5**; *p* > 0.05). Indeed, the content of DHA in the liver was positively correlated to the dietary inclusion levels of the combination of pungent spices (Pearson correlation coefficient *r* = 0.72, *p* = 0.008). Likewise, the total levels of n-3 polyunsaturated fatty acids (n-3 PUFAs) in the liver followed a similar trend, increasing with higher concentrations of pungent spices in the diets (*r* = 0.73, *p* = 0.007). The fatty acid profile of fillet was also very conserved, although the DHA/EPA ratio significantly increased in fish fed the SPICY_0.1%_ diet (**Table 6**; *p* < 0.05).

**Table 5:** Fatty acid profile (mg/g lipid) of the liver in gilthead seabream fed the control and two basal diets supplemented with a mixture of pungent spices (capsicum, black pepper, ginger, and cinnamaldehyde) at a dietary inclusion level of 0.1 (SPICY_0.1%_) and 0.15% (SPICY_0.15%_).

	Control	SPICY_0.1%_	SPICY_0.15%_
Myristic acid (C14:0)	8.61 ± 2.87	6.96 ± 2.62	8.66 ± 1.59
Pentadecylic acid (C15:0)	1.47 ± 0.29	1.35 ± 0.31	1.27 ± 0.25
Palmitic acid (C16:0)	125.93 ± 2.91	117.43 ± 9.82	132.13 ± 9.26
Stearic acid (C18:0)	46.63 ± 2.66	50.24 ± 6.98	52.10 ± 5.64
Saturated fatty acids (SFAs)	176.52 ± 19.53	179.57 ± 13.97	197.27 ± 12.73
Palmitoleic acid (C16:1 n-7)	26.20 ± 4.93	24.56 ± 2.82	28.42 ± 1.74
Vaccenic acid (C18:1 n-7)	34.41 ± 3.06	33.67 ± 8.12	35.80 ± 5.39
Oleic acid (C18:1 n-9)	238.38 ± 8.25	246.14 ± 35.72	261.72 ± 30.28
Eicosenoic acid (C20:1 n-9)	4.08 ± 0.39	5.06 ± 1.96	4.43 ± 0.77
Nervionic acid (C24:1 n-9)	1.76 ± 0.15	1.99 ± 0.37	1.93 ± 0.33
Monounsaturated fatty acids (MUFAs)	307.14 ± 13.72	311.83 ± 44.12	332.29 ± 36.43
Linoleic acid (C18:2 n-6)	126.14 ± 6.76	124.04 ± 10.12	138.17 ± 9.49
Gamma-linolenic acid (C18:3 n-6)	6.94 ± 1.24	7.84 ± 2.32	7.10 ± 0.77
Arachidonic acid (C20:4 n-6; ARA)	4.47 ± 0.73	5.02 ± 0.73	5.47 ± 0.37
n-6 polyunsaturated fatty acids (n-6 PUFAs)	137.90 ± 5.82	136.90 ± 12.21	150.74 ± 9.92
Alpha-linolenic acid (C18:3 n-3)	8.48 ± 0.35	8.06 ± 0.53	8.99 ± 0.38
Stearidonic acid (C18:4 n-3)	1.52 ± 0.04	1.43 ± 0.26	1.73 ± 0.22
Eicosatetraenoic acid (C20:4 n-3)	1.67 ± 0.22	1.75 ± 0.13	1.81 ± 0.16
Eicosapentaenoic acid (C20:5 n-3; EPA)	15.17 ± 0.39	15.48 ± 1.39	16.96 ± 1.80
Docosapentaenoic acid (C22:5 n-3)	7.96 ± 1.10	9.02 ± 1.93	8.92 ± 0.49
Docosahexaenoic acid (C22:6 n-3; DHA)	15.85 ± 2.21^a^	17.77 ± 0.63^ab^	20.80 ± 2.58^b^
n-3 polyunsaturated fatty acids (n-3 PUFAs)	49.15 ± 4.88^a^	53.65 ± 2.72^ab^	59.21 ± 4.61^b^
Total PUFAs	189.48 ± 5.85	190.55 ± 14.73	209.95 ± 14.45
DHA/EPA	1.11 ± 0.09	1.15 ± 0.07	1.23 ± 0.09
EPA + DHA	32.05 ± 0.81	33.24 ± 1.95	37.76 ± 4.15
n6/n3	2.67 ± 0.12	2.55 ± 0.12	2.55 ± 0.06

Non-represented fatty acids were not detected in the analysis. Values are represented as mean ± SD (n = 4 tanks per dietary group) and differences among groups (p ≤ 0.05) are indicated by the different superscript letters.

**Table 6:** Fatty acid profile (mg/g lipid) of the fillet in gilthead seabream fed the control and two basal diets supplemented with a mixture of pungent spices (capsicum, black pepper, ginger, and cinnamaldehyde) at a dietary inclusion level of 0.1 (SPICY_0.1%_) and 0.15% (SPICY_0.15%_).

	Control	SPICY_0.1%_	SPICY_0.15%_
Myristic acid (C14:0)	8.60 ± 1.35	8.34 ± 1.59	8.72 ± 1.88
Pentadecylic acid (C15:0)	1.21 ± 0.10	1.14 ± 0.12	1.14 ± 0.11
Palmitic acid (C16:0)	129.29 ± 3.49	125.17 ± 5.42	128.34 ± 9.20
Stearic acid (C18:0)	32.41 ± 1.39	32.20 ± 1.07	32.70 ± 0.64
Lignoceric acid (C24:0)	1.44 ± 0.16	1.49 ± 0.05	1.48 ± 0.28
Saturated fatty acids (SFAs)	173.63 ± 2.79	169.19 ± 7.07	172.77 ± 11.93
Palmitoleic acid (C16:1 n-7)	33.50 ± 2.27	31.87 ± 2.63	34.05 ± 5.60
Oleic acid (C18:1 n-9)	247.55 ± 10.03	239.58 ± 12.32	249.47 ± 23.17
Eicosenoic acid (C20:1 n-9)	3.39 ± 0.14	3.51 ± 0.35	3.26 ± 0.39
Nervionic acid (C24:1 n-9)	1.43 ± 0.22	1.54 ± 0.11	1.58 ± 0.14
Monounsaturated fatty acids (MUFAs)	285.59 ± 12.55	276.50 ± 13.65	287.93 ± 28.38
Linoleic acid (C18:2 n-6)	143.89 ± 7.49	141.64 ± 5.88	143.51 ± 11.97
Gamma-linolenic acid (C18:3 n-6)	3.26 ± 0.37	3.44 ± 0.63	3.34 ± 0.12
Arachidonic acid (C20:4 n-6; ARA)	5.03 ± 0.43	5.17 ± 0.34	5.06 ± 0.96
n-6 polyunsaturated fatty acids (n-6 PUFAs)	152.56 ± 8.17	150.26 ± 6.44	151.91 ± 11.40
Alpha-linolenic acid (C18:3 n-3)	9.82 ± 0.78	9.41 ± 0.75	9.92 ± 0.89
Stearidonic acid (C18:4 n-3)	1.56 ± 0.15	1.60 ± 0.25	1.64 ± 0.24
Eicosatetraenoic acid (C20:4 n-3)	1.63 ± 0.23	1.65 ± 0.15	1.58 ± 0.17
Eicosapentaenoic acid (C20:5 n-3; EPA)	23.87 ± 2.38	22.58 ± 0.48	23.71 ± 3.64
Docosapentaenoic acid (C22:5 n-3)	9.92 ± 1.22	9.60 ± 0.13	9.32 ± 0.55
Docosahexaenoic acid (C22:6 n-3; DHA)	29.37 ± 2.75	30.83 ± 1.58	30.37 ± 4.95
n-3 polyunsaturated fatty acids (n-3 PUFAs)	77.88 ± 9.41	75.68 ± 2.29	76.39 ± 8.28
Total PUFAs	230.43 ± 15.81	225.94 ± 8.28	228.30 ± 12.78
DHA/EPA	1.23 ± 0.03^a^	1.37 ± 0.08^b^	1.28 ± 0.06^ab^
EPA + DHA	53.24 ± 5.08	53.41 ± 1.62	54.07 ± 8.50
n6/n3	1.97 ± 0.18	1.99 ± 0.06	2.01 ± 0.29

Non-represented fatty acids were not detected in the analysis. Values are represented as mean ± SD (n = 4 tanks per dietary group), and differences among groups (p ≤ 0.05) are indicated by the different superscript letters.

### 3.3 Hepatic metabolism, oxidative stress biomarkers, and activity of pancreatic digestive enzymes

Dietary supplementation with the combination of pungent spices did not alter the specific activity of the measured metabolic or oxidative stress enzymes, nor were there changes in LPO and TAC in the liver (**Supplementary Table S5**; *p* > 0.05). Regarding pancreatic digestive enzymes, there were no significant differences in the specific activities of total alkaline proteases, bile-salt-activated lipase, and α-amylase among diets in the stomach and pyloric ceca samples (**Table 7**; *p* > 0.05). On the other hand, the activity of bile-salt-activated lipase increased in the AI in gilthead seabream fed both supplemented diet with respect to the control group (*p* < 0.05), whereas the activities of total alkaline proteases and α-amylase followed the same numerical trend, but differences were not significant due to large interindividual variability (*p* > 0.05).

**Table 7:** Specific activity (mU/mg protein) of total alkaline proteases, α-amylase, and bile-salt-activated lipase in gilthead seabream fed the control and two basal diets supplemented with a mixture of pungent spices (capsicum, black pepper, ginger, and cinnamaldehyde) at a dietary inclusion level of 0.1 (SPICY_0.1%_) and 0.15% (SPICY_0.15%_).

	Control	SPICY_0.1%_	SPICY_0.15%_
Stomach and pyloric caeca			
Total alkaline proteases	78.52 ± 8.01	90.20 ± 15.61	89.73 ± 12.03
α-amylase	398.32 ± 51.08	410.19 ± 25.24	418.92 ± 30.81
Bile salt-activated lipase	21.49 ± 6.26	23.48 ± 1.54	22.90 ± 4.83
Anterior intestine			
Total alkaline proteases	133.64 ± 13.45	145.24 ± 11.82	160.35 ± 25.63
α-amylase	402.98 ± 130.44	492.51 ± 80.53	475.20 ± 60.72
Bile salt-activated lipase	50.00 ± 9.57^a^	65.19 ± 4.84^b^	63.94 ± 2.81^b^

Values are represented as mean ± SD (n = 4 tanks per dietary group) and differences among groups (p ≤ 0.05) are indicated by the different superscript letters.

### 3.4 Composition of bile

Two primary BAs in their tauro-conjugated form were detected in the bile of gilthead seabream: the taurocholic acid (T-CA) and the taurochenodeoxycholic acid (T-CDCA), both at similar levels among dietary groups (**Table 8**; *p* > 0.05). While the concentration of BAs did not change among dietary treatments in the gallbladder, the numerical values in the AI of each group notably varied even though this trend was not statistically significant due to large interindividual variability (*p* > 0.05). In particular, the mean levels of T-CA and T-CDCA increased 50% and 20%, respectively, in fish fed the SPICY_0.15%_ diet, with respect to the control diet. That resulted in an increase of more than 35% of the mean levels of total BAs, although statistically there were no significant differences due to the high deviation among specimens within each dietary group.

**Table 8:** Bile acid profile in the gallbladder and the anterior intestine in gilthead seabream fed the control and two basal diets supplemented with a mixture of pungent spices (capsicum, black pepper, ginger, and cinnamaldehyde) at a dietary inclusion level of 0.1 (SPICY_0.1%_) and 0.15% (SPICY_0.15%_).

	Control	SPICY_0.1%_	SPICY_0.15%_
Gallbladder (mg/mL)			
T-CA	108.23 ± 9.59	108.89 ± 1.11	100.23 ± 8.14
T-CDCA	47.67 ± 3.69	46.76 ± 5.14	46.76 ± 9.04
Total BAs	155.90 ± 9.58	155.65 ± 5.62	146.98 ± 15.27
Anterior intestine (µg/mg)			
T-CA	30.00 ± 15.83	44.44 ± 13.05	34.32 ± 12.06
T-CDCA	19.94 ± 13.72	23.90 ± 6.18	17.26 ± 4.26
Total BAs	49.94 ± 29.41	68.34 ± 19.07	51.58 ± 16.28

Non-represented bile acids were not detected in the analysis. Values are represented as mean ± SD (n = 4 tanks per dietary group). T-CA, taurocholic acid; T-CDCA, taurochenodeoxycholic acid; total BAs, total bile acids.

### 3.5 Histomorphology of the liver and anterior intestine

**Figure 1:**
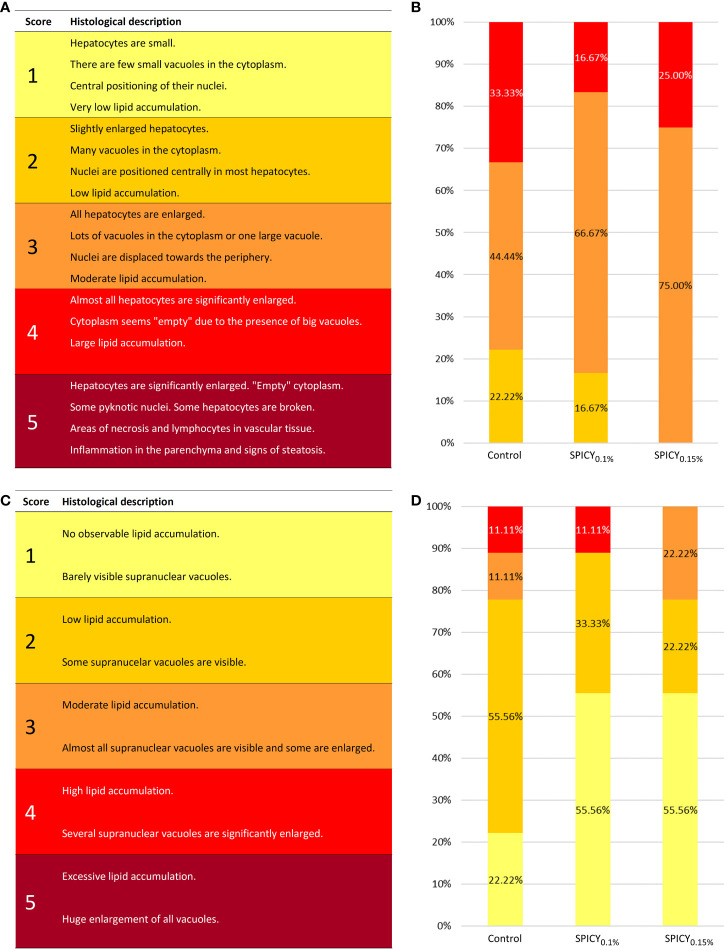
**(A)** Semi-quantitative scoring used for evaluating the levels of fat accumulation in the liver of gilthead seabream [adapted from Ruiz et al. (31)]. **(B)** Results (%) of hepatic scoring from gilthead seabream (n = 12 fish per dietary group) fed the control and two basal diets supplemented with a mixture of pungent spices (capsicum, black pepper, ginger, and cinnamaldehyde) at a dietary inclusion level of 0.1 (SPICY_0.1%_) and 0.15% (SPICY_0.15%_). **(C)** Semi-quantitative scoring used for evaluating the levels of fat accumulation in the anterior intestine of gilthead seabream [adapted from Ruiz et al. (31)]. **(D)** Results (%) of intestinal scoring from gilthead seabream (n = 12 fish per dietary group) fed the experimental diets.

Under the present experimental conditions, the hepatic parenchyma showed a typical histological organization in all dietary groups. In particular, hepatocytes were polyhedral in shape, with varying degrees of vacuolization in their cytoplasm, and arranged in anastomosed plates separated by sinusoidal capillaries leading to central veins. No signs of inflammation nor infiltration of lymphocytes were observed in any of the diets. The SPICY_0.1%_ diet had a clear effect on the accumulation of fat deposits within hepatocytes, halving the number of individuals with high lipid accumulation in liver (score classification of 4) and increasing the number of those with low and moderate lipid accumulation (classifications 2 and 3; **Figures 1A, B** **)**.

The histological organization of AI was also typical, without signs of enteritis. In brief, the mucosa was lined with the columnar epithelial layer, supported by connective tissue of the lamina propria-submucosa and surrounded by the tunica muscularis. The epithelium was mainly composed of enterocytes with acidic microvilli, a basal basophilic nucleus, eosinophilic cytoplasm, and different amount and size of clear supranuclear vacuoles depending on the dietary treatment. In particular, both diets supplemented with the mixture of pungent spices were able to reduce vacuolization with respect to the control group (**Figures 1C, D** **)**. No differences in villus or enterocyte height, density of goblet cells, and thickness of the tunica muscularis were found among treatments (**Supplementary Table S6**; *p* > 0.05).

### 3.6 Microbial diversity, structure, and composition

Based on the rest of the results, only the control and SPICY_0.1%_ diets were selected for the microbiota and gene expression analyses.

**Figure 2:**
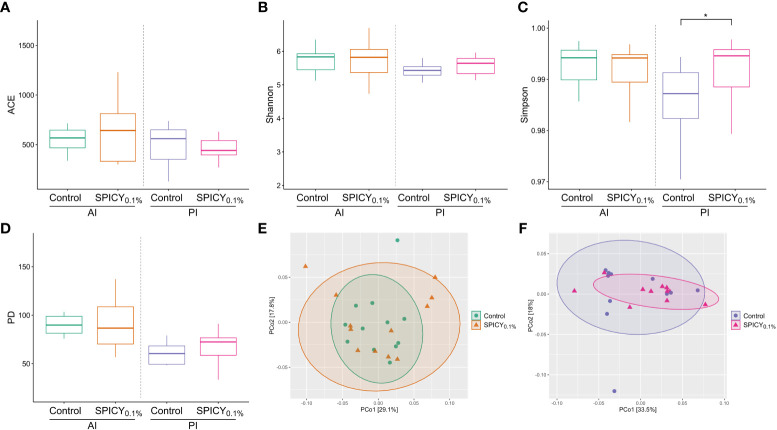
Microbial alpha diversity in the anterior (AI) and posterior intestine (PI) in gilthead seabream (n = 12 fish per dietary group) fed the control and the basal diet supplemented with a mixture of pungent spices (capsicum, black pepper, ginger, and cinnamaldehyde) at a dietary inclusion level of 0.1% (SPICY_0.1%_): **(A)** ACE index, **(B)** Shannon index, **(C)** Simpson’s index, **(D)** Faith’s phylogenetic diversity (PD); and PCoA analyses showing the spatial distribution of microbiota samples from **(E)** the AI and **(F)** the PI based on weighted UniFrac distances. Asterisks represent significant differences between dietary treatments (*p* ≤ 0.05).

After rarefaction, a total of 2,269,502 reads clustering into 19,379 ASVs were obtained. The alpha diversity indices of ACE, Shannon, and Faith’s phylogenetic diversity (PD) were not different among dietary treatments (control vs. SPICY_0.1%_) regardless of the region of the intestine considered (**Figures 2A, B, D**; *p* > 0.05), while in the PI, the values from Simpson’s Diversity Index increased in fish fed the SPICY_0.1%_ diet (0.99 ± 0.00; mean ± SEM) in comparison to the control group (0.96 ± 0.02) (**Figure 2C**; *p* < 0.05; **Supplementary Table S7**). Results of beta diversity based on the weighted UniFrac analysis did not show separation among specimens regarding diets in the AI (**Figure 2E**; PERMANOVA, *F* = 0.666, *R* ^2 = ^0.029, *p* = 0.827) nor in the PI (**Figure 2F**; PERMANOVA, *F* = 0.950, *R* ^2 = ^0.045, *p* = 0.408).

**Figure 3:**
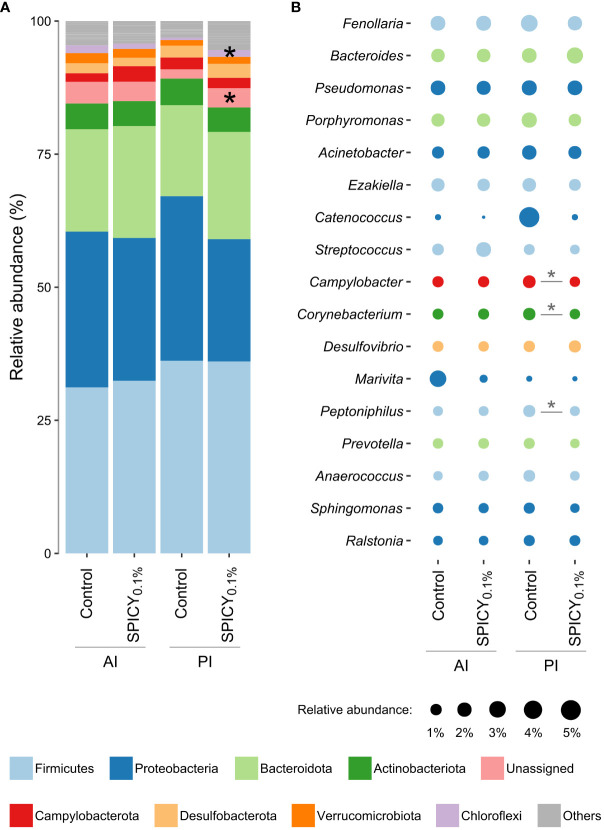
Relative abundances of gut bacterial taxa in the anterior (AI) and posterior intestine (PI) in gilthead seabream (n = 12 fish per dietary group) fed the control and the basal diet supplemented with a mixture of pungent spices (capsicum, black pepper, ginger, and cinnamaldehyde) at a dietary inclusion level of 0.1% (SPICY_0.1%_). Data are expressed at **(A)** phylum and **(B)** genus levels (excluding unassigned genera). Taxa appearance in the figures is in order of decreasing abundance (from bottom to top in the bar graph, and inversely in the bubble plot). Taxa with an abundance <1% are classified as others in the bar graph and not represented in the bubble plot. Asterisks represent significant differences between dietary treatments (*p* ≤ 0.05).

In terms of microbial composition, the most abundant phyla were Firmicutes, Proteobacteria, and Bacteroidota, accounting for 81% of the total microbial population (**Figure 3A**). There were no significant differences in the relative abundance of any of these three phyla among dietary treatments (**Supplementary Table S8**; *p* > 0.05). On the other hand, there was a significant increase in the phylum Chloroflexi in the PI in fish fed the SPICY_0.1%_ diet with respect to the control group (*p* < 0.05). The Firmicutes/Bacteroidetes (F/B) ratio was maintained at 1.67–1.70 in the AI and at 1.92–2.13 in the PI (*p* > 0.05). Similarly, the relative abundances of the most dominant genera (≥1.0%) did not change among diets in the AI (**Figure 3B**; **Supplementary Table S9**; *p* > 0.05). Nevertheless, the relative abundances of the genera *Campylobacter*, *Corynebacterium*, and *Peptoniphilus* in the PI decreased when adding the combination of pungent spices to the diet (*p* < 0.05).

### 3.7 Gene expression profile of the liver and anterior intestine

**Figure 4:**
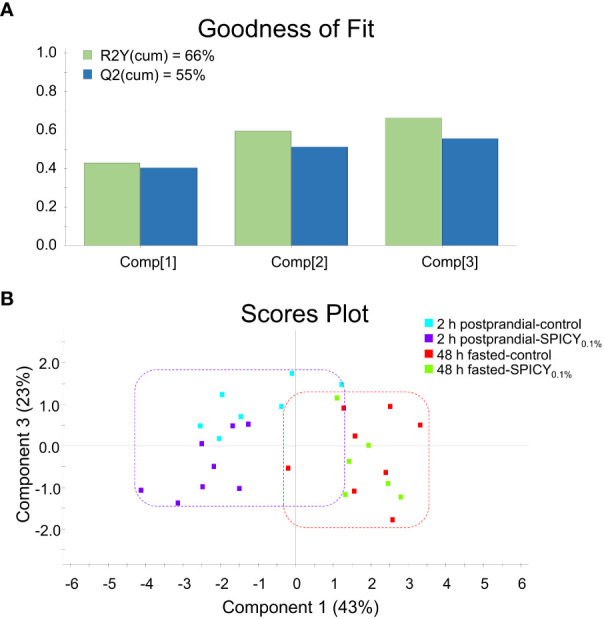
**(A)** Goodness of fit of the PLS-DA model. **(B)** Score plot for two-dimensional PLS-DA representing individual distribution between the component 1 and 3 of the model based on hepatic biomarker expression values with *p* ≤ 0.1 (Student’s t-test) among diets. Samples were obtained from the liver of 48-h fasted and 2-h postprandial gilthead seabream (n = 8 fish per dietary group) fed the control and the basal diet supplemented with a mixture of pungent spices (capsicum, black pepper, ginger, and cinnamaldehyde) at a dietary inclusion level of 0.1% (SPICY_0.1%_).

The expression patterns of 33 out of 44 genes analyzed in the liver were affected by the feeding time (2 h postprandial vs. 48 h fasted) (**Supplementary Table S10**; *p* < 0.1). Analysis of differences among diets for each feeding time highlighted an upregulation of *fasn*, *elovl6*, and *cyp7a1* (*p* < 0.05) and to a lower degree of *scd1b* (*p* < 0.1), while *lpl* (*p* < 0.05) and *pparβ* (*p* < 0.1) were downregulated in 2-h postprandial fish fed the SPICY_0.1%_ diet with respect to those fed the control diet. In 48-h fasted animals, experimental diets only affected *srebp1* and *prdx5* expression patterns, which were upregulated in fish fed the SPICY_0.1%_ diet (*p* < 0.05). The PLS-DA model of liver expression was based on three components, with an explained variance [R2Y(cum)] of 66% and a predicted variance [Q2(cum)] of 55% (**Figure 4A**). Separation among individuals regarding the different assayed feeding times (2 h postprandial vs. 48 h fasted) was not clear, nor was there a differential distribution of individuals based on diet (**Figure 4B**).

**Figure 5:**
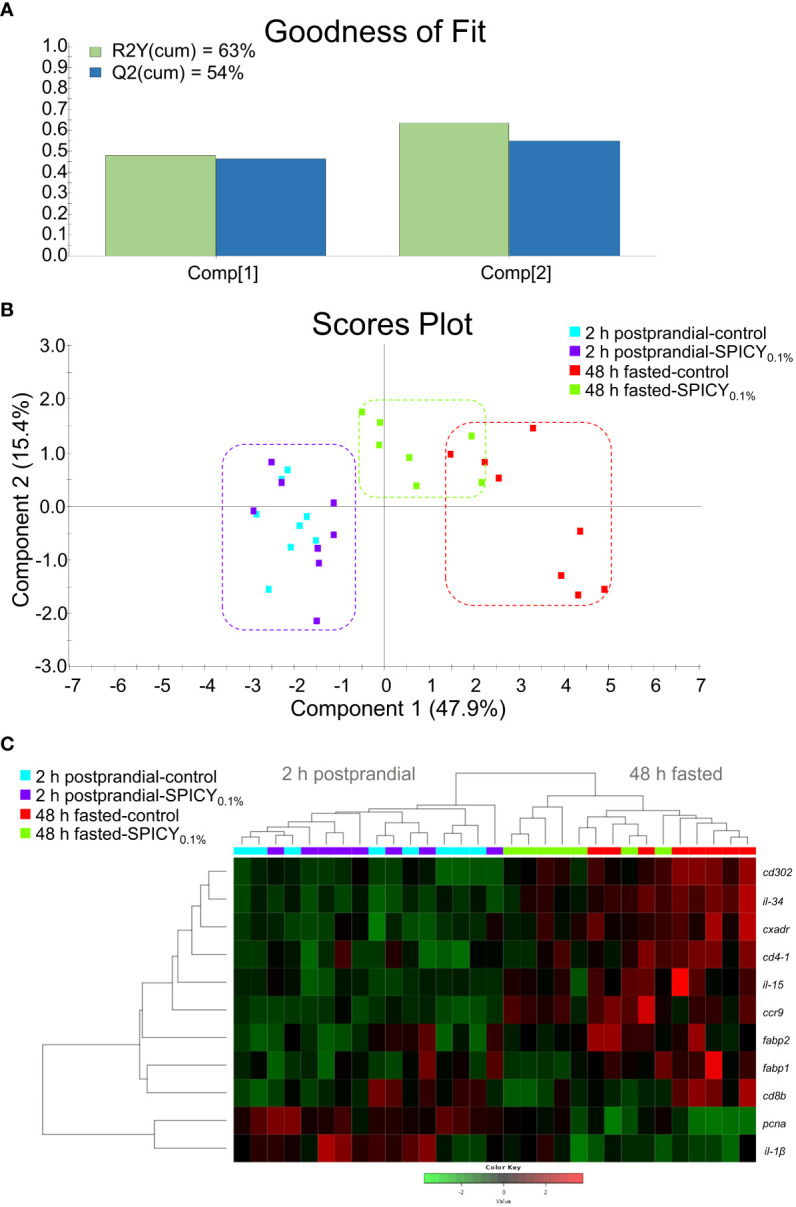
**(A)** Goodness of fit of the PLS-DA model. **(B)** Scores plot for two-dimensional PLS-DA representing individual distribution between the two components of the model based on intestinal biomarker expression values with *p* ≤ 0.1 (Student’s t-test) among diets. **(C)** Heatmap plotting hierarchical clustering of intestinal biomarker expression values (color key scale) with *p* ≤ 0.1. Samples were obtained from the anterior intestine of 48-h fasted and 2-h postprandial gilthead seabream (n = 8 fish per dietary group) fed the control and the basal diet supplemented with a mixture of pungent spices (capsicum, black pepper, ginger, and cinnamaldehyde) at a dietary inclusion level of 0.1% (SPICY_0.1%_).

In the AI, feeding time caused major changes on the expression of 29 out of the 44 analyzed genes (**Supplementary Table S11**; *p* < 0.1). Regarding diet differences at each feeding time, the SPICY_0.1%_ diet only upregulated *il-1β* and *ccr9* with respect to the control 2-h postprandial fish (Student’s t-test, *p* < 0.1), whereas after the 48-h fasting period, the SPICY_0.1%_ diet caused a significant downregulation of *fabp1*, *fabp2*, *il-34*, *cd4-1*, *cd8b*, and *cd302* (*p* < 0.05) and, to a lesser extent, *cxadr* and *il-15*, and an upregulation of *pcna* (*p* < 0.1). The PLS-DA model of the AI expression was based on two components, with an R2Y(cum) of 63% and a Q2(cum) of 54% (**Figure 5A**). There was a clear separation among specimens regarding feeding time along component 1, which explained 47.9% of the total variance (**Figure 5B**), although there were no differences among individuals for 2-h postprandial fish, and a certain overlap among individuals fed the control and SPICY_0.1%_ diets in 48-h fasted fish. Consequently, clustering was able to discriminate feeding time but not diet differences at each feeding time (**Figure 5C**).

## 4 Discussion

Present results revealed that the combination of capsicum, black pepper, and ginger oleoresins, and cinnamaldehyde promoted somatic growth in gilthead seabream (BW_f_ and SGR) at both dietary levels tested (0.1% and 0.15%). Such results are partially in accordance with those obtained when testing the same combination of pungent spices in broiler chickens, with production phase differences that can be attributed to inherent differences in species-specific physiology, production system, diet formulation, among other factors (41). In fish species, there is still scarce information on the pungent spices herein tested. However, a recent study was conducted in gilthead seabream under winter farming conditions (16 ± 2°C) and fed diets with a high content of SFAs (40%; 16% crude fat), in which the same blend of pungent spices was tested at different inclusion levels of 0.05%, 0.1%, and 0.15% (20). In this study, somatic growth presented a positive dose–response trend, but effects were not significant.

To our knowledge, there are no other studies in fish species testing the combined effect of all four spices evaluated herein. Nonetheless, the effects of capsicum, black pepper, and ginger (or their active principles) have been separately assessed on fish performance, with controversial results (**Supplementary Table S12**). In this sense, some studies have reported that the dietary inclusion of capsicum did not lead to a significant improvement in BW nor SGR in gilthead seabream, blue streak hap (*Labidochromis caeruleus*), Mozambique tilapia (*Oreochromis mossambicus*), rainbow trout (*Oncorhynchus mykiss*), and jewel cichlid (*Hemichromis guttatus*) (56–60). On the other hand, Talebi et al. (61) showed that the dietary supplementation of red bell pepper (*Capsicum annum*) at an inclusion level of 44 or 55 mg/kg for 20–60 days significantly increased the BW and total length, but not the SGR in rainbow trout (*Oncorhynchus mykiss*). When supplementing fish diets with black pepper or its extracts, growth was not enhanced in African catfish (*Clarias gariepinus*), rainbow trout, and common carp (*Cyprinus carpio*) (62–64), but weight gain was significantly increased in rohu fish (*Labeo rohita*) when adding the extract to the diet at an inclusion level of 1% or 2% (65). Among the four spices tested in the current study, ginger has been the most studied regarding its growth enhancing effect in teleosts (66–70, among others). Reviewing the literature, different results have been reported, even in the same species, depending on the study. Such changes in growth performance may be attributed to the specific conditions and design of each nutritional assay, namely, feeding period duration, basal diet formulation, supplement dosage, and form (e.g., dried powder, essential oil, or oleoresin), and to the physiological condition of the fishes. In the case of cinnamaldehyde, despite the absence of differences in the growth of Nile tilapia (*Oreochromis niloticus*) when supplementing the diets with this active principle (1 and 2 mL/kg) reported by Amer et al. (71), many studies have demonstrated its potential in improving growth performance in different fish species, such as Nile tilapia, grass carp (*Ctenopharyngodon idella*), tongue sole (*Cynoglossus semilaevis*), and fat greenling (*Hexagrammos otakii*) (72–75).

Regarding somatic indices, the fact that there were no significant differences in Fulton’s condition factor among dietary treatments suggested that the tested combination of pungent spices did not compromise the overall body condition of gilthead seabream juveniles (76). Furthermore, the HSI was also not altered, in line with the absence of inflammation upon histological evaluation and the absence of differences in the activity of hepatic metabolic stress and oxidative stress enzymes, which confirmed the good health condition of the liver. On the other hand, PVFI was significantly decreased by more than 20% in fish fed the SPICY_0.1%_ diet. In this regard, the diminishment of the perivisceral fat levels usually has a positive effect on the consumer’s perception (i.e., visual impact, more pleasant smell) and extends the shelf-life of the edible fraction (77, 78). Despite the reduced PVFI, the proximate macronutrient composition and fatty acid profile of the liver and fillet in gilthead seabream fed the SPICY_0.1%_ diet were very conserved.

When supplementing the diet with the combination of pungent spices at 0.15%, the hepatic levels of DHA and n-3 PUFAs significantly increased. These results are highly remarkable, since n3-PUFAs, and especially DHA, are essential to ensure optimal fish growth, development, and reproduction, and to enhance their immune response (4). Regarding the fillet, the DHA/EPA ratio was significantly increased in fish fed the SPICY_0.1%_ diet in comparison to the control group, reaching values closer to those expected in gilthead seabream juveniles fed conventional diets without fish oil replacement (79, 80). This was associated with a slight numerical increase in DHA and decrease in EPA levels in the fillet of fish fed the SPICY_0.1%_ diet. However, the reason why the combination of pungent spices increased the DHA/EPA ratio is presently unclear. In the previous study testing the same combination of spices in gilthead seabream (20), DHA/EPA ratio in fillet was not significantly affected, and both DHA and EPA were numerically increased by the combination of pungent spices (although DHA had a higher numerical increase and, hence, DHA/EPA was slightly raised). The main effect previously described was a significant increase in the n-3 PUFA levels in fillet and a numerical reduction in n-6 PUFA in seabream fed a diet supplemented with 0.15% of the spicy additive (20; Patent Number WO/2022/117810). Since gilthead seabream has a restricted ability to synthesize DHA and EPA (4), it is unlikely that the observed differences are caused by changes in LC-PUFA biosynthesis pathways. Supporting this, the expression of the genes encoding the enzymes that elongate PUFAs (*elovl4*, *elovl5*, and probably *elovl1*; 81) was not upregulated in fish fed the SPICY_0.1%_ diet, although the higher increase in hepatic levels of DHA was observed in fish fed the SPICY_0.15%,_ diet, for which gene expression was not assayed. Another possibility is that the increase in the levels of n-3 PUFAs could be caused by a higher capacity of absorption and assimilation of n-3 PUFAs (specifically DHA) from the diet, conferred by the combination of pungent spices, and related to the increased bile-salt-activated lipase activity, as further discussed below. However, a more likely hypothesis is that these fatty acids were better conserved and deposited in fish tissues. There is good evidence suggesting that animal fats can promote LC-PUFA sparing, associated with their high SFA content, which are preferentially used as metabolic energy sources (82, 83). On the other hand, spices, including the ones used in the combination tested herein, are well-known promotors of fat oxidation in mammals (84). Hence, the combination of pungent spices could have further potentiated the oxidation of dietary SFAs and MUFAs for energy purposes and the sparing of LC-PUFAs, with its resulting increased tissue deposition. Furthermore, in line with the present results, DHA has been shown to be more efficiently retained than EPA in Atlantic salmon tissues (85), which has been explained by the higher complexity of DHA catabolism as compared to EPA, since DHA oxidation requires an extra step involving the peroxisomes (86).

Under the current experimental conditions, the supplementation of the diet with the combination of pungent spices at 0.1% reduced the FCR, in spite of the absence of significant differences in feed intake and lipid and protein ADCs. A similar decrease in FCR was reported in the study of Morais et al. (20) when supplementing gilthead seabream diets with 0.1% and 0.15% of this additive during winter. While none of the aforementioned studies testing the effect of capsicum in fish diets described an amelioration of FCR (56, 58–60), some of the works with black pepper or ginger and many of those with cinnamaldehyde supplementation did (63, 65, 66, 68, 70, 72, 74, 75). Therefore, the decrease in FCR in fish fed the SPICY_0.1%_ diet might be partly attributed to the active principles of ginger and black pepper and to cinnamaldehyde or to the synergetic effect of the four spices present in the tested product. One of the possible mechanisms by which the combination of pungent spices decreased FCR values may lie, at least partly, in the induction of the bile-salt-activated lipase in the AI, as its activity was higher when supplementing the diets with the tested combination.

When evaluating the histological organization of the liver and AI, none of the fish presented an excessive fat accumulation (score of 5—steatosis; **Figure 1**), nor were there signs of inflammation or physiological alterations, the presence of which normally indicates disorders associated with unbalanced dietary conditions (87). The most effective dose of the tested additive for reducing hepatic lipid deposits was 0.1%, while in the intestine, both inclusion levels followed a similar dynamic in terms of regulation of intracellular fat deposits. In this sense, it is well-established in mammals that several different spices have an effect on the regulation of body adiposity through different mechanisms (18, 19). One of these mechanisms is the stimulation of lipid digestion, which may occur through two different pathways, as discussed in Platel and Srinivasan (18). The first is by stimulating the activity of digestive pancreatic and intestinal enzymes, whereas the second pathway is associated with the potential of the spices to induce a higher secretion of BAs from the liver into the bile, and subsequently to the intestine. Bile acids allow fat emulsification and provide a higher action surface for lipases through the formation of micelles, and being necessary for the activation of bile-salt-activated lipase (88). In the current experiment, we did not report statistical variation on lipid ADC values among experimental groups, which might be related to the relatively high water temperature (22.5°C ± 0.5°C), not imposing a major challenge for lipid digestibility. Nevertheless, the numerical increase in the levels of BAs (T-CA, T-CDCA, and their sum) in the AI of fish fed the SPICY_0.1%_ diet and the higher activity of the bile-salt-activated lipase at both dietary inclusion levels indicated a stimulatory effect towards hepatic production and/or secretion of BAs by the tested combination of spices. To our knowledge, this is currently the only study that has evaluated the fish BA profile under dietary supplementation with any of the spices herein tested. Drawing on the existing mammalian literature, some studies on rats have reported an induced synthesis and secretion of BAs into bile after dietary supplementation with capsaicin (the pungent active principle of capsicum) or ginger, the latter also increasing the bile flowrate. By contrast, dietary supplementation with piperine (the active principle of black pepper) and with cinnamon (the main source of cinnamaldehyde) did not show a stimulatory effect on BA synthesis and secretion (18, 19). However, when the four spices were combined in a mixture, along with others, and supplemented in rat diets, the secretion of BAs and bile flowrate markedly increased, even though the higher increase was shown when the mixture did not contain cinnamon (89). In the current study, the apparently increased secretion of BAs may be associated with a higher synthesis of BAs in the liver, considering the significantly upregulated expression of cholesterol 7-alpha-monooxygenase (*cyp7a1*) in fish fed the SPICY_0.1%_ diet. Indeed, CYP7A1 is the first rate-limiting enzyme in the classic pathway of the biosynthesis of BAs from cholesterol.

Considering the results on growth and feeding performance, DHA/EPA ratio of the fillet, vacuolization levels in the liver and AI, the activity of bile-salt-activated lipase, and the BA profile, the best dietary inclusion level of the combination of pungent spices under the present experimental conditions was 0.1%. Hence, samples from the control and SPICY_0.1%_ diets fed fish were selected for analyzing the gene expression patterns of the liver and AI, and the gut microbial communities. Assuming a higher synthesis and secretion of BAs in fish fed the SPICY_0.1%_ diet, these BAs might be responsible for the 2-h postprandial hepatic downregulation of lipoprotein lipase (*lpl*) and upregulation of fatty acid synthase (*fasn*). In this regard, we previously carried out an assay feeding gilthead seabream juveniles with the same basal diet used in this study but supplemented with a blend of BAs (31), and the expression patterns of *lpl* and *fasn* followed similar trends to the ones of the current work. Regarding LPL, this enzyme hydrolyzes the triacylglycerides from chylomicrons and very low-density lipoproteins circulating in the plasma into glycerol and free fatty acids (90). In this way, LPL facilitates the posterior incorporation of these fatty acids into the tissues, which are stored as an energetic reservoir in the form of triacylglycerides. Thus, *lpl* downregulation may be in line with the decreased PVFI and lower levels of fat deposits in the liver. On the other hand, FASN is a key lipogenic enzyme involved in *de novo* fatty acid synthesis, which catalyzes the conversion of malonyl-CoA and acetyl-CoA into palmitic (C16:0) acid (90). Furthermore, Dorn et al. (91) suggested a correlation between *fasn* expression and hepatocellular lipid accumulation. Thus, the upregulation of *fasn* could be a counter-regulatory mechanism to maintain a balance on the hepatic fat deposits and on the levels of fatty acids regarding their lower incorporation caused by reduced expression of *lpl* (31). In this sense, many studies in fish have reported an inverse regulation between the expression patterns of *lpl* and *fasn* in the liver (92–94, among others). It might be worthwhile to further examine the mechanisms underlying the transcription of these two biomarkers of lipid metabolism in fish from a deeper molecular perspective. Interestingly, in the current study, the supplementation of the diet with the combination of pungent spices also led to a hepatic upregulation of the *de novo* lipogenic biomarkers stearoyl-CoA desaturase 1b (*scd1b*) and elongation of very long chain fatty acids 6 (*elovl6*). SCD1 metabolizes palmitic and stearic (C18:0) acid into palmitoleic (C16:1 n-7) and oleic (C18:1 n-9) acid, respectively (95), while ELOVL6 elongates SFAs and MUFAs with 12, 14, and 16 carbons (96). Assuming that activity and gene expression matched, the upregulation of *elovl6* and *scd1b* may be part of the mechanism, in which *fasn* is involved, to stabilize hepatic fatty acid levels in response to their lower incorporation linked to *lpl* downregulation and/or be a strategy to prevent accumulation of palmitic (C16:0) acid triggered by *fasn* upregulation. The expression changes in the abovementioned biomarkers of lipid metabolism were accompanied by the downregulation of peroxisome proliferator-activated receptor β (*pparβ*) in fish fed the SPICY_0.1%_ diet. The role of PPARβ in lipid metabolism is not yet fully elucidated, and the change reported in this work was not very pronounced given its low expression levels. However, PPARβ may stimulate the expression of genes involved in fatty acid oxidation, and its ligands are likely fatty acids (97, 98), so a possibility is that *pparβ* is downregulated due to the presumed lower hepatic accumulation of fatty acids linked to *lpl* downregulation, which would also match the lower PVFI observed in fish fed the SPICY_0.1%_ diet.

Regarding the gut microbial composition, several studies have highlighted that the most dominant bacterial phyla of the gut microbial communities in gilthead seabream are Firmicutes, Proteobacteria, Bacteroidota, and Actinobacteriota (44, 99), in line with the results obtained in the present work. Under current conditions, the gut microbial community composition was very conserved, with only an increase in the relative abundance of Chloroflexi in the PI of fish fed the SPICY_0.1%_ diet. This is a widespread and metabolically diverse phylum of bacteria that has been reported as part of fish microbiota (44, 100, 101), but whose role remains unknown. No differences were found in the values of the F/B ratio, which is a well-documented factor whose changes have been used as a biomarker of intestinal dysbiosis in fishes (102). Our F/B values remain within the results reported for farmed fish, which vary widely depending on the rearing conditions and experimental diets utilized (103, 104). On the other hand, the absence of differences in beta diversity (Weighted UniFrac) indicated that all samples were similar in terms of their phylogenetic features, and therefore, the addition of pungent spices to the diet did not pose a threat in terms of dysbiosis. Regarding alpha diversity metrics, there were no differences in estimated richness (Chao1 and ACE indices) or phylogenetic diversity (Faith index), but there was an increased value of Simpson’s diversity index in the PI of fish fed the SPICY_0.1%_ diet with respect to the control group. Both the Shannon and Simpson’s indices are estimators of species richness and evenness, but while the former puts more weight on species richness, the latter puts more weight on species evenness (51). Therefore, the variation reported with Simpson’s Index is more likely due to the differential abundances of species among dietary treatments. Such changes in abundance may be attributed to the potential antimicrobial effect on some bacterial strains of the spices present in the additive (105–108). Specifically, in the current study, there was a reduction in relative abundance of the genera *Campylobacter*, *Corynebacterium*, and *Peptoniphilus* in the PI using the pungent spices supplement at a dietary inclusion level of 0.1%. On the other hand, such changes in abundance may also be related to the presumed higher secretion of bile stimulated by the spices and/or to the higher levels of BAs found in the intestine of fish fed the SPICY_0.1%_ diet. Furthermore, when supplementing the diets with the combination of pungent spices in the present work, there was an increase in the relative abundance of unassigned genera belonging to the family Lachnospiraceae (**Supplementary Table S9**), some members of whom possess bile acids-inducible genes, which encode for enzymes involved in the metabolism of primary BAs into secondary BAs (109). In addition, *Prevotella*, which is a non-bile-acid-resistant genus, tended to be present at a lower relative abundance in fish fed the SPICY_0.1%_ diet than the control group (*p* = 0.051), which can be seen as another evidence of an increase in the secretion of bile acids.

As clearly reflected by the PLS-DA representation, the overall gene expression profile from the intestine of fed animals (2 h postprandial) did not show any significant differences among dietary groups (**Supplementary Table S11**). Only the expressions of interleukin-1 beta (*il-1β*) and C–C chemokine receptor type 9 (*ccr9*) showed a substantial increase in fish fed the SPICY_0.1%_ diet (*p* < 0.1). IL-1β is a pro-inflammatory cytokine produced by several cell types in response to different processes, such as the activation of pattern recognition receptors (PRRs) by pathogen-associated molecular patterns (PAMPs) or danger-associated molecular patterns (DAMPs), among others. Additionally, IL-1β is responsible for a cascade of effects on different members of this cytokine family, leading to signal transduction and activation of the nuclear factor (NF)-kB pathway (110). Furthermore, CCR9 is a cell marker found in a wide range of immune cells (B cells, T cells, monocytes, macrophages, and dendritic cells) that drives their migration to the gut-associated lymphoid tissue (GALT), where these cells may play a regulatory role in inflammation (111). Considering the absence of signs of intestinal inflammation under histological analysis and the absence of expression changes on other pro-inflammatory cytokines (*tnf-α*, *il-6*, *il-8*, *il-15*, or *il-34*) and on the measured PRRs, the upregulation of *il-1β* and *ccr9* may be attributed to a mild innate immune system priming. Since CCR9 has both pro- and anti-inflammatory functions (111), it is difficult to know whether the upregulation of *ccr9* was also associated with the pro-inflammatory effect that IL-1β may have on the intestine, or it was part of an anti-inflammatory response to maintain homeostasis counteracting IL-1β-induced pro-inflammatory response. In any case, as mentioned above, no signs of inflammation or physiological disorders were observed with any of the dietary treatments under current experimental conditions, meaning that during the feeding period, the supplementation of pungent spices did not compromise the intestinal health of the fish.

Regarding the gene expression of biomarkers of inflammation in 48-h fasted fish, an anti-inflammatory effect of the tested additive was indicated by the downregulation of interleukin-15 (*il-15*), interleukin-34 (*il-34*), cluster of differentiation 4-1 (*cd4-1*), cluster of differentiation 8 beta (*cd8b*), and CD302 antigen (*cd302*). While the role of IL-15 and IL-34 in the fish immune response is yet unclear, many studies have suggested that, as in mammals, these cytokines may be associated with an inflammatory response (110). In mammals, IL-15 induces the proliferation of B cells, T cells, and natural killer (NK) cells (112), and IL-34 stimulates the proliferation and differentiation of monocytes and macrophages and can promote the proliferation of CD8^+^ T regulatory cells (113). Thus, the downregulation of both cytokines may be in line with the lower expression of the cell markers *cd4* and *cd8b*, which are commonly found on the surface of different T-cell subtypes (114). Similarly, the C-type lectin receptor CD302 is expressed in monocytes, macrophages, granulocytes, and dendritic cells and is involved in cell adhesion and migration and in endocytosis and phagocytosis (115). Some *in vitro* assays using cells from ayu (*Plecoglossus altivelis*) and Nile tilapia have also unraveled the phagocytic and bactericidal activity of CD302 (116, 117). In this sense, the downregulation of *il-15*, *il-34*, *cd4-1*, *cd8b*, and *cd302* may be related to an anti-inflammatory effect of the tested combination of pungent spices. This has been seen previously when each of the spices was tested individually in mammals (107, 118) and is also in line with the anti-inflammatory effect that ginger and cinnamaldehyde apparently have in the intestine of rohu fish and bacteria-infected zebrafish (*Danio rerio*), respectively (68, 119). Although black pepper and capsicum have also been reported to act as immunostimulants in fish, such as rainbow trout and rohu fish, respectively (65, 120, 121), their effects on inflammation have not yet been elucidated.

In addition to the potential anti-inflammatory response, the dietary supplementation with the combination of pungent spices may also have an effect on intestinal epithelial integrity. In particular, there was an upregulation of proliferating cell nuclear antigen (*pcna*), which may suggest a higher cell proliferation (122) and, in line with it, a downregulation of the coxsackievirus and adenovirus receptor homolog (*cxadr*), whose expression has been inversely correlated with the rate of cell proliferation (123). Based on these discussed results, a possibility is that such enhanced functionality may modulate the potential entry of PAMPs through the enterocytes, which could be the cause of the lower expression of the PRR *cd302* by the leukocytes of the GALT. Consequently, this would lead to a lower immune response as indicated by the lower expression of *cd4-1* and *cd8b* cell markers and reduced secretion of the pro-inflammatory cytokines IL-15 and IL-34. Hence, the reduced levels of expression of pro-inflammatory biomarkers may be the consequence of a reduced exposure to foreign molecules due to the increased permeability induced by *pcna* upregulation rather than an orchestrated activation of an anti-inflammatory response. Regardless of the cause of the downregulation of *il-15*, *il-34*, *cd4-1*, *cd8b*, and *cd302*, the results suggest that the tested additive enhanced the health status of the intestine under short-term fasting conditions, since the SPICY_0.1%_ diet prevented intestinal inflammation that can affect the fish intestinal motility and deregulate digestion and absorption of nutrients, leading to a lower utilization of the feed and to physiological disorders (124). In this sense, some strategies currently used in aquaculture, such as the replacement of fish oil or the use of high-fat diets, have been correlated with inflammation and excessive lipid accumulation in digestive tissues and with a lower non-specific immunity and disease resistance (16, 125, 126). Therefore, the tested combination of pungent spices may be used as a potential tool to aid in the prevention of intestinal inflammation, enhance peristalsis, and optimize nutrient digestion and absorption. However, further studies are needed to confirm the anti-inflammatory effect in the intestine of the combination of spices tested herein. Overall, to complete the detailed understanding of the mechanisms underlying the above-mentioned pathways, the invaluable help of omics sciences (i.e., RNA-seq or microarray analyses) may be needed in order to further track the gene expression of key biomarkers, such as some anti-inflammatory cytokines (TGF-β, IL-4/13A, and IL-4/13B).

Although there was no suggestion of potential anti-inflammatory effect of the combination of pungent spices until feeding cessation, this was probably because the transit of feed along the gastrointestinal tract had a greater impact on gene expression than the differential ingredient formulation of the experimental diets themselves. Regardless, based on our above-discussed results, we recommend that the incorporation of the combination of pungent spices in the diet be continuous whenever the purpose is to promote lipid metabolism to reduce body fat content and improve the health status of the fish, accompanied by an increase in fish growth and feed utilization. In this sense, the regulatory effect that the combination of spices had on the expression of hepatic biomarkers related to lipid metabolism practically disappeared after the 48-h fasting period (**Supplementary Table S10**), except for the upregulation of sterol regulatory element-binding proteins 1 (*srebp1*) and peroxiredoxin 5 (*prdx5*). SREBP1 is a membrane transcription factor that has been related to lipid homeostasis through the regulation of *de novo* lipogenesis and fatty acid synthesis in fish liver (81), which may be activated after feed deprivation to maintain a balance on the levels of hepatic fatty acids. Albeit the function of PRDX5 in fish still needs to be further studied, recent works in mammals have elucidated its role in preventing adipogenesis by maintaining the intracellular redox balance and avoiding fat deposition through inhibition of fatty acid synthesis and acceleration of their oxidation (127, 128). Thus, the decreased fatty acid production triggered by *prdx5* upregulation in the liver of 48-h fasted fish fed the SPICY_0.1%_ diet may be in line with the downregulation in the intestine of the liver-type fatty acid-binding protein (*fabp1*) and the intestinal fatty acid-binding protein (*fabp2*) (**Supplementary Table S11**), both involved in fatty acid uptake, transport, and metabolism (129).

## 5 Conclusions

This study showed that under the regimen of a high saturated fat diet (with poultry fat as the major lipid source), the supplementation of the feed with 0.1% of a product containing a combination of capsicum, black pepper, and ginger oleoresins, and cinnamaldehyde improved the growth performance and FCR in gilthead seabream. In addition, the SPICY_0.1%_ diet reduced the levels of fat deposits in the visceral cavity, liver, and intestine and increased the DHA/EPA ratio in the fillet to a range closer to the levels commonly found in fillet of farmed gilthead seabream fed a conventional fish-oil-based diet. Moreover, the SPICY_0.1%_ diet was able to increase the activity of the bile-salt-activated lipase and regulate the gene expression of biomarkers of lipid metabolism. In this sense, the upregulation of *cyp7a1*, which is involved in the synthesis of primary BAs, which may stimulate lipid digestion, and the downregulation of *lpl*, which hydrolyzes plasmatic triacylglycerides releasing free fatty acids that can be incorporated into the tissues, was noteworthy. There was no great impact in the structure and composition of the gut microbial communities, albeit the relative abundance of the phylum Chloroflexi increased, while the relative abundances of the genera *Campylobacter*, *Corynebacterium*, and *Peptoniphilus* decreased. In addition, when supplementing the basal diet with the combination of pungent spices at 0.1%, there was a downregulation of the PRR *cd302*, the immune cell markers *cd4-1* and *cd8b*, and the pro-inflammatory cytokines *il-15* and *il-34* in 48-h fasted fish. On the other hand, the supplementation of the diet with the pungent spices at an inclusion level of 0.15% also had a positive effect on growth performance, in the reduction in lipid vacuolization within the enterocytes, and in the activity of the bile-salt-activated lipase in the intestine. Moreover, the SPICY_0.15%_ diet showed higher levels of DHA and total n-3 PUFAs in the liver than the control group. Therefore, the supplementation of fish feeds containing reduced levels of fish oil, especially when fish oil is substituted by oils containing high levels of SFAs, with the combination of pungent spices, may provide a functional solution to optimize the usage of fish oil as a strategic ingredient in the aquafeed industry, even though the optimal inclusion level might need to be optimized depending on the species and diet composition.

## Data availability statement

The datasets presented in this study can be found in online repositories. The names of the repository/repositories and accession number(s) can be found in the article/**Supplementary Material.**

## Ethics statement

The animal study was approved by Ethical Committee of the Institute of Agrifood Research and Technology and the Generalitat of Catalunya. The study was conducted in accordance with the local legislation and institutional requirements.

## Author contributions

SM and EG conceptualized the research. EG designed the nutritional assay. AR, IS, PH, and JP processed the samples. AR, PH, JC-G, JP, MV, JP-S, and EG analyzed the data. AR, KA, DF, JC-G, JP, JP-S, SM, and EG interpreted the results. AR wrote the original draft of the manuscript. EG supervised the study and the writing of the original draft and acquired the funding. All authors contributed to the article and approved the submitted version.
