# Supplementary material for: The potential of a combination of pungent spices as a novel supplement in gilthead seabream (Sparus aurata) diets to aid in the strategic use of fish oil in aquafeeds: a holistic perspective
Source: Front Immunol. 2023 Sep 25;14:1222173. doi: 10.3389/fimmu.2023.1222173 (PMC10561386; doi:10.3389/fimmu.2023.1222173)

Supplementary Material

Supplementary Table 1. Fatty acid composition (mg/g lipid) of experimental diets: a control and two basal diets supplemented with a mixture of pungent spices (capsicum, black pepper, ginger and cinnamaldehyde) at a dietary inclusion level of 0.1% ($SPICY_{0.1\%}$) and 0.15% ($SPICY_{0.15\%}$).

	**Control**	**$SPICY_{0.1\%}$**	**$SPICY_{0.15\%}$**
Myristic acid (C14:0)	9.58 ± 0.37	9.65 ± 0.10	9.00 ± 1.00
Pentadecylic acid (C15:0)	1.44 ± 0.09	1.45 ± 0.10	1.44 ± 0.02
Palmitic acid (C16:0)	117.17 ± 2.46	115.64 ± 0.54	120.59 ± 6.83
Stearic acid (C18:0)	31.57 ± 0.51	32.69 ± 0.61	35.15 ± 0.42
Saturated fatty acids (SFAs)	163.40 ± 2.40	163.25 ± 0.07	168.99 ± 8.30
Palmitoleic acid (C16:1 n-7)	26.49 ± 0.54	25.49 ± 0.34	26.13 ± 0.92
Oleic acid (C18:1 n-9)	190.26 ± 4.18	182.44 ± 3.18	191.27 ± 2.14
Eicosenoic acid (C20:1 n-9)	2.41 ± 0.10	2.19 ± 0.02	2.23 ± 0.02
Monounsaturated fatty acids (MUFAs)	220.00 ± 4.39	211.30 ± 3.48	220.27 ± 3.10
Linoleic acid (C18:2 n-6)	153.85 ± 0.04	151.18 ± 2.90	155.27 ± 3.12
Gamma-linolenic acid (C18:3 n-6)	1.95 ± 0.29	1.50 ± 0.32	1.63 ± 0.01
Arachidonic acid (C20:4 n-6; ARA)	4.34 ± 0.06	4.13 ± 0.05	4.15 ± 0.06
n-6 polyunsaturated fatty acids (n-6 PUFAs)	160.14 ± 0.39	156.82 ± 2.63	161.04 ± 3.17
Alpha-linolenic acid (C18:3 n-3)	11.70 ± 0.41	11.66 ± 0.07	11.96 ± 0.03
Stearidonic acid (C18:4 n-3)	2.26 ± 0.24	2.85 ± 0.16	2.98 ± 0.01
Eicosatetraenoic acid (C20:4 n-3)	0.94 ± 0.03	0.76 ± 0.13	0.93 ± 0.05
Eicosapentaenoic acid (C20:5 n-3; EPA)	24.14 ± 1.23	22.86 ± 0.33	24.23 ± 0.37
Docosapentaenoic acid (C22:5 n-3)	3.25 ± 0.16	3.35 ± 0.11	3.23 ± 0.32
Docosahexaenoic acid (C22:6 n-3; DHA)	14.29 ± 0.25	13.87 ± 0.24	14.46 ± 0.02
n-3 polyunsaturated fatty acids (n-3 PUFAs)	57.39 ± 2.33	56.10 ± 0.20	58.50 ± 0.02
Total PUFAs	217.53 ± 2.71	212.92 ± 2.82	219.54 ± 3.14

Non represented fatty acids were not detected in the analysis. Values are represented as mean ± SD. Fatty acid composition of diets was analysed in duplicate.

Supplementary Table 2. Gilthead seabream primers for qPCR amplification of hepatic genes.

Gene	Symbol	GenBank	Primer
Fatty acid synthase	*fasn*	JQ277708	F: ACA GGC AGC GTC GGT GCC AGT GGT CTA C<br>R: TCC AGG ACG CAG CCT GCC GCG AAC TAC
Elongation of very long chain fatty acids 1	*elovl1*	JX975700	F: CTT CCT ACA CAT CTT CCA CCA CTC<br>R: CCA TTC CAC CAG GAG CAA AGG
Elongation of very long chain fatty acids 4	*elovl4*	JX975701	F: CGG TGG CAA TCA TCT TCC<br>R: TCA ACT GGC TGT CTG TGT
Elongation of very long chain fatty acids 5	*elovl5*	AY660879	F: CCT CCT GGT GCT CT ACA AT<br>R: GTG AGT GTC CTG GCA GTA
Elongation of very long chain fatty acids 6	*elovl6*	JX975702	F: GTG CTG CTC TAC TCC TGG TA<br>R: ACG GCA TGG ACC AAG TAG T
Fatty acid desaturase 2	*fads2*	AY055749	F: GCA GGC GGA GAG CGA CGG TCT GTT CC<br>R: AGC AGG ATG TGA CCC AGG TGG AGG CAG AAG
Stearoyl-CoA desaturase 1a	*scd1a*	JQ277703	F: CGG AGG CGG AGG CGT TGG AGA AGA AG<br>R: AGG GAG ACG GCG TAC AGG GCA CCT ATA TG
Stearoyl-CoA desaturase 1b	*scd1b*	JQ277704	F: GCT CAA TCT CAC CAC CGC CTT CAT AG<br>R: GCT GCC GTC GCC CGT TCT CTG
Cholesterol 7-alpha-monooxygenase	*cyp7a1*	KX122017	F: CCC TGC TAT TAA AGT CCC ACC TCT<br>R: ATC GTA GGT AGG CTG GAG GAT TC
Phospholipid transfer protein	*pltp*	XM_030418561	F: TTG TGT CCT CCA TCA TGT CCA GC<br>R: TTC AAC ATC TCC AGT CCT CTA TCA GTG A
Adipose triglyceride lipase	*atgl*	JX975711	F: GTG CTT CAG TCC TGG ATG TCT TC<br>R: AGC CTT GCA GGT CCA TGT TGA
Hepatic lipase	*hl*	EU254479	F: TTG TAG AAG GTG AGG AAA ACT G<br>R: GCT CTC CAT CAG ACC ATC C
Lipoprotein lipase	*lpl*	AY495672	F: CGT TGC CAA GTT TGT GAC CTG<br>R: AGG GTG TTC TGG TTG TCT GC
85kDa calcium-independent phospholipase A2	*pla2g6*	JX975708	F: CGC CAA GGA ACT CGG AAA GAT GCT<br>R: ACC GCA CAG CCA TCA GAG TCT
Hepatocyte nuclear factor 4 alpha	*hnf4a*	FJ360721	F: TCG GAG GTT CTG CCA ATG AG<br>R: TGC TGA GGT GCT CCT GAA C
Sterol regulatory element-binding proteins 1	*srebp1*	JQ277709	F: AGG GCT GAC CAC AAC GTC TCC TCT CC<br>R: GCT GTA CGT GGG ATG TGA TGG TTT GGG
Sterol regulatory element-binding protein 2	*srebp2*	XM_030408996	F: TCA GCA GGC TCT ACA CAG AAC<br>R: GAA CGG CAG GTC GTG AGA
Farnesoid X receptor	*fxr*	XM_030426192	F: CTG GAG GTC TAT GAC GCT GAG AT<br>R: CAC ACG GCA GGC ATC ACA
Liver X receptor α	*lxrα*	FJ502320	F: GCA CTT CGC CTC CAG GAC AAG<br>R: CAG TCT TCA CAC AGC CAC ATC AGG
Peroxisome proliferator-activated receptor α	*pparα*	AY590299	F: TCT CTT CAG CCC ACC ATC CC<br>R: ATC CCA GCG TGT CGT CTC C
Peroxisome proliferator-activated receptor β	*pparβ*	AY590301	F: AGG CGA GGG AGA GTG AGG ATG AGG AG<br>R: CTG TTC TGA AAG CGA GGG TGA CGA TGT TTG
Peroxisome proliferator-activated receptor γ	*pparγ*	AY590304	F: CGC CGT GGA CCT GTC AGA GC<br>R: GGA ATG GAT GGA GGA GGA GGA GAT GG
Carnitine palmitoyltransferase 1A	*cpt1a*	JQ308822	F: GTG CCT TCG TTC GTT CCA TGA TC<br>R: TGA TGC TTA TCT GCT GCC TGT TTG
Hydroxyacyl-CoA dehydrogenase	*hadh*	JQ308829	F: GAA CCT CAG CAA CAA GCC AAG AG<br>R: CTA AGA GGC GGT TGA CAA TGA ATC C
Fatty acid translocase/CD36	*fat/cd36*	XM_030440140	F: GAC CAG AGG AGG ACC ACA TC<br>R: GTT GTC GGA GTG AGT GAA TAA GC
Fatty acid binding protein, heart	*h-fabp*	JQ308834	F: CTG GGT GTG GGC TTC GCT AC<br>R: CTC TGT GTT CTT GAT GGT GCT CTG
Citrate synthase	*cs*	JX975229	F: TCC AGG AGG TGA CGA GCC<br>R: GTG ACC AGC AGC CAG AAG AG

NADH-ubiquinone oxidoreductase chain 2	*nd2*	KC217558	F: TAG GTT GAA TGA CCA TCG TA<br>R: GGC TAA GGA GTT GAG GTT
NADH-ubiquinone oxidoreductase chain 5	*nd5*	KC217559	F: CCT AAA CGC CTG AGC CCT GG<br>R: GCT GTA AAC GAG GTG GCT AGA AGG
Cytochrome c oxidase subunit I	*coxi*	KC217652	F: GTC CTA CTT CTT CTG TCC CTT CCT GTT CT<br>R: AGG TTT CGG TCT GTA AGG AGC ATT GTA ATC
Proliferator-activated receptor gamma coactivator 1 alpha	*pgc1α*	JX975264	F: CGT GGG ACA GGT GTA ACC AGG ACT C<br>R: ACC AAC CAA GGC AGC ACA CTC TAA TTC T
Sirtuin1	*sirt1*	KF018666	F: GGT TCC TAC AGT TTC ATC CAG CAG CAC ATC<br>R: CCT CAG AAT GGT CCT CGG ATC GGT CTC
Sirtuin2	*sirt2*	KF018667	F: GAA CAA TCC GAC GAC AGC AGT GAA G<br>R: AGG TTA CGC AGG AAG TCC ATC TCT
Catalase	*cat*	JQ308823	F: TGG TCG AGA ACT TGA AGG CTG TC<br>R: AGG ACG CAG AAA TGG CAG AGG
Uncoupling protein 1	*ucp1*	FJ710211	F: GCA CAC TAC CCA ACA TCA CAA G<br>R: CGC CGA ACG CAG AAA CAA AG
Glutathione peroxidase 1	*gpx1*	DQ524992	F: GAA GGT GGA TGT GAA TGG AAA AGA TG<br>R: CTG ACG GGA CTC CAA ATG ATG G
Glutathione peroxidase 4	*gpx4*	AM977818	F: TGC GTC TGA TAG GGT CCA CTG TC<br>R: GTC TGC CAG TCC TCT GTC GG
Peroxiredoxin 3	*prdx3*	GQ252681	F: ATC AAC ACC CCA CGC AAG ACT G<br>R: ACC GTT TGG ATC AAT GAG GAA CAG ACC
Peroxiredoxin 5	*prdx5*	GQ252683	F: GAG CAC GGA ACA GAT GGC AAG G<br>R: TCC ACA TTG ATC TTC TTC ACG ACT CC
Superoxide dismutase [Cu-Zn]	*cu-zn-sod / sod1*	JQ308832	F: TCA CGG ACA AGA TGC TCA CTC TC<br>R: GGT TCT GCC AAT GAT GGA CAA GG
Superoxide dismutase [Mn]	*mn-sod / sod2*	JQ308833	F: CCT GAC CTG ACC TAC GAC TAT GG<br>R: AGT GCC TCC TGA TAT TTC TCC TCT G
Glucose-regulated protein, 170 kDa	*grp-170*	JQ308821	F: CAG AGG AGG CAG ACA GCA AGA C<br>R: TTC TCA GAC TCA GCA TTT CCA GAT TTC
Glucose-regulated protein, 94 kDa	*grp-94*	JQ308820	F: AAG GCA CAG GCT TAC CAG ACA G<br>R: CTT CAG CAT CAT CGC CGA CTT TC
Glucose-regulated protein, 75 kDa	*grp-75*	DQ524993	F: TCC GGT GTG GAT CTG ACC AAA GAC<br>R: TGT TTA GGC CCA GAA GCA TCC ATG
Beta-actin	*actb*	X89920	F: TCC TGC GGA ATC CAT GAG A<br>R: GAC GTC GCA CTT CAT GAT GCT

Supplementary Table 3. Gilthead seabream primers for qPCR amplification of intestinal genes.

Gene	Symbol	GenBank	Primer
Proliferating cell nuclear antigen	*pcna*	KF857335	F: CGT ATC TGC CGT GAC CTG T<br>R: AGA ACT TGA CTC CGT CCT TGG
Transcription factor HES-1-B	*hes1-b*	KF857344	F: GCC TGC CGA TAT GAT GGA A<br>R: GGA GTT GTG TTC ATG CTT GC
Krüppel-like factor 4	*klf4*	KF857346	F: ACA TCA CCG CAC GCA CAC<br>R: AAC CAC AGC CCT CCC AGT C
Claudin-12	*cldn12*	KF861992	F: CTC TCA GGG CTA CAC ATC TAC CTA TGC<br>R: ACA TTC GTG AGC GGC TGG AG
Claudin-15	*cldn15*	KF861993	F: CCG ATT GTG GAA GTA GTG GCT CTG GT<br>R: CAG CAT CAC CCA ACC GAC GAA CC
Cadherin-1	*cdh1*	KF861995	F: TGC TCC ATA CAG CGT CAC CTT ACA<br>R: CTC GTT CAT CCT AGC CGT CCA GTT
Cadherin-17	*cdh17*	KF861996	F: GAT GCC CGC AAC CCA GAG<br>R: CCG TTG ATT CAC TGC CGT AGA C
Tight junction protein ZO-1	*tjp1*	KF861994	F: AAG CAG TAT TAC GGT GAC TCA<br>R: TGC ATC CCT GGC TTG TAG
Desmoplakin	*dsp*	KF861999	F: GCA GAA GGA GCA CGA GAC CATC<br>R: GGG TGT TCT TGT CGC AGG TGA A
Gap junction Cx32.2 protein	*cx32.2*	KF862000	F: CGA GGT GTT CTA TCT GCT CTG TA<br>R: CTT GTG GGT GCG AGT CCT
Coxsackievirus and adenovirus receptor homolog	*cxadr*	KF861998	F: CAT CAG AGG ACT ACG AGA GG<br>R: CAT CTT GGC AGC ATT TGG T
Intestinal-type alkaline phosphatase	*alpi*	KF857309	F: CCG CTA TGA GTT GGA CCG TGA T<br>R: GCT TTC TCC ACC ATC TCA GTA AGG G
Liver type fatty acid-binding protein	*fabp1*	KF857311	F: GTC CTC GTC AAC ACC TTC ACC AT<br>R: CGC CTT CAT CTT CTC GCC AGT
Intestinal fatty acid-binding protein	*fabp2*	KF857310	F: CGA GCA CAT TCC GCA CCA AAG<br>R: CCC ACG CAC CCG AGA CTT C
Ileal fatty acid-binding protein	*fabp6*	KF857312	F: ACC CAG GAC GGC AAT ACC<br>R: CGA CGG TGA AGT TGT TGG T
Mucin 2	*muc2*	JQ277710	F: ACG CTT CAG CAA TCG CAC CAT<br>R: CCA CAA CCA CAC TCC TCC ACA T
Mucin 13	*muc13*	JQ277713	F: TTC AAA CCC GTG TGG TCC AG<br>R: GCA CAA GCA GAC ATA GTT CGG ATA T
Tumor necrosis factor-alpha	*tnf-α*	AJ413189	F: CAG GCG TCG TTC AGA GTC TC<br>R: CTG TGG CTG AGA GCT GTG AG
Interleukin-1 beta	*il-1β*	AJ419178	F: GCG ACC TAC CTG CCA CCT ACA CC<br>R: TCG TCC ACC GCC TCC AGA TGC
Interleukin-6	*il-6*	EU244588	F: TCT TGA AGG TGG TGC TGG AAG TG<br>R: AAG GAC AAT CTG CTG GAA GTG AGG
Interleukin-7	*Il-7*	JX976618	F: CTA TCT CTG TCC CTG TCC TGT GA<br>R: TGC GGA TGG TTG CCT TGT AAT
Interleukin-8	*il-8*	JX976619	F: CAG CAG AGT CTT CAT CGT CAC TAT TG<br>R: AGG CTC GCT TCA CTG ATG G
Interleukin-10	*il-10*	JX976621	F: AAC ATC CTG GGC TTC TAT CTG<br>R: GTG TCC TCC GTC TCA TCT G
			F: ATT CCC TGT GTG GTG GCT GCT

Interleukin-12 subunit beta	*il-12β*	JX976624	R: GCT GGC ATC CTG GCA CTG AAT
Interleukin-15	*il-15*	JX976625	F: GAG ACC AGC GAG CGA AAG GCA TCC<br>R: GCC AGA ACA GGT TAC AGG TTG ACA GGA A
Interleukin-34	*il-34*	JX976629	F: TCT GTC TGC CTG CTG GTA G<br>R: ATG CTG GCT GGT GTC TGG
CD4-1	*cd4-1*	AM489485	F: TCCTCCTCCTCGTCCTCGTT<br>R: GGTGTCTCATCTTCCGCTGTCT
CD8 beta	*cd8b*	KX231275	F: CCGAAATGTGGAAGACTGGAACTC<br>R: CTTTGGAGGTAAGGTTGGAGGGAT
C-C chemokine receptor type 3	*ccr3*	KF857317	F: CTA CAT CAG CAT CAC CAT ACG CAT CCT<br>R: TGG CAC GGC ACT TCT CCT TCA
C-C chemokine receptor type 9	*ccr9*	KF857318	F: TCC CTG AGT TAA TCT TCG CCC AAG TG<br>R: TGT TGT ATT CGT TGT TCC AGT AGA CCA GAG
C-C chemokine receptor type 11	*ccr11*	KF857319	F: GCT ACG ATT ACA GTT ATG AA<br>R: TAG ATG ATT GGG AGG AAG
C-C chemokine CK8 / C-C motif chemokine 20	*ck8/ ccl20*	GU181393	F: CCG TCC TCA TCT GCT TCA TAC T<br>R: GCT CTG CCG TTG ATG GAA C
Macrophage colony-stimulating factor 1 receptor 1	*csf1r1*	AM050293	F: TTG CGT GTG GTG AGG AAG GAA GGT<br>R: AGC AGG CAG GGC AGC AGG TA
Immunoglobulin M	*igm*	JQ811851	F: ACC TCA GCG TCC TTC AGT GTT TAT GAT GCC<br>R: CAG CGT CGT CGT CAA CAA GCC AAG C
Immunoglobulin T membrane-bound form	*igt-m*	KX599201	F: AGA CGA TGC CAG TGA AGA GGA TGA GT<br>R: CGA AGG AGG AGG CTG TGG ACC A
Galectin-1	*lgals1*	KF862003	F: GTG TGA GGA GGT CCG TGA TG<br>R: ACT GTA GAG CCG TCC GAT AGG
Galectin-8	*lgals8*	KF862004	F: GGC GGT GAA CGG CGG TCA<br>R: GCT CCA GCT CCA GTC TGT GTT GAT AC
Toll-like receptor 2	*tlr2*	KF857323	F: CAT CTG CGA CTC TCC TCT CTT CCT<br>R: ATT CAA CAA TGG AGC GGT GGA CTT
Toll-like receptor 5	*tlr5*	KF857324	F: TCG CCA ATC TGA CGG ACC TGA G<br>R: CAG AAC GCC GAT GTG GTT GTA AGA C
Toll-like receptor 9	*tlr9*	AY751797	F: GCC TTC CTT GTC TGC TCT TTC T<br>R: GCC GTA GAG GTG CTT CAG TAG
CD209 antigen-like protein D	*cd209d*	KF857327	F: CGC CAC GAG CAT GAG GAC AA<br>R: TCT TGC CAG AAT CCA TCA CCA TCC A
CD302 antigen	*cd302*	KF857328	F: GGA CCA GAG GAA GAG CAC ATC<br>R: GAC CAG GGC GGA CAT CAG
Macrophage mannose receptor 1	*mrc1*	KF857326	F: CTT CCG ACC GTA CCT GTA CCT ACT CA<br>R: CGA TTC CAG CCT TCC GCA CAC TTA
Fucolectin	*fcl*	KF857331	F: CCA TAC TGC TGA ACA GAC CAA CC<br>R: TGA TGG AGG TGA CGA TGT AGG A
Beta-Actin	*actb*	X89920	F: TCCTGCGGAATCCATGAGA<br>R: GACGTCGCACTTCATGATGCT

Supplementary Table 4. Proximate composition (%) of dry mass from the liver and the fillet of gilthead seabream fed the control and two basal diets supplemented with a mixture of pungent spices (capsicum, black pepper, ginger and cinnamaldehyde) at a dietary inclusion level of 0.1% ($SPICY_{0.1\%}$) and 0.15% ($SPICY_{0.15\%}$).

	Control	$SPICY_{0.1\%}$	$SPICY_{0.15\%}$
**Liver**			
Protein (%)	21.72 ± 1.35	22.18 ± 3.42	23.40 ± 0.58
Lipid (%)	42.62 ± 3.12	37.68 ± 4.17	40.40 ± 3.71
Carbohydrates (%)	24.39 ± 2.47	20.13 ± 2.63	24.10 ± 4.56
Ash (%)	2.70 ± 0.19	2.63 ± 0.46	2.73 ± 0.38
**Fillet**			
Protein (%)	79.81 ± 0.78	80.62 ± 0.49	80.23 ± 0.75
Lipid (%)	12.97 ± 1.75	12.04 ± 1.42	14.93 ± 3.85
Carbohydrates (%)	0.98 ± 0.15	0.97 ± 0.09	0.85 ± 0.15
Ash (%)	6.96 ± 0.42	7.40 ± 0.94	7.07 ± 0.69

Values are represented as mean ± SD (n = 4 tanks per dietary group).

Supplementary Table 5. Activity of hepatic enzymes and oxidative condition of gilthead seabream fed the control and two basal diets supplemented with a mixture of pungent spices (capsicum, black pepper, ginger and cinnamaldehyde) at a dietary inclusion level of 0.1% ($SPICY_{0.1\%}$) and 0.15% ($SPICY_{0.15\%}$).

	Control	$SPICY_{0.1\%}$	$SPICY_{0.15\%}$
**Hepatic Metabolism**			
LDH (mU mg protein^{-1})	75.49 ± 19.98	76.93 ± 4.72	69.16 ± 15.65
AST (mU mg protein^{-1})	812.56 ± 180.70	811.46 ± 133.55	782.10 ± 176.44
ALT (mU mg protein^{-1})	251.12 ± 37.33	318.23 ± 22.65	306.33 ± 38.06
**Oxidative Stress in Liver**			
GR (nmol min^{-1} mg protein^{-1})	3.22 ± 0.73	3.61 ± 0.60	3.55 ± 0.80
CAT (nmol min^{-1} mg protein^{-1})	96.88 ± 5.62	90.43 ± 13.26	95.61 ± 9.00
SOD (% enzyme inhibition)	56.58 ± 9.23	58.26 ± 9.49	61.47 ± 10.66
LPO (nmol MDA/g)	15.52 ± 3.66	12.87 ± 2.00	14.83 ± 2.64
Trolox equivalents (nmol/µL)	18.50 ± 1.72	19.14 ± 2.01	21.04 ± 1.87

Values are represented as mean ± SD (n = 4 tanks per dietary group).

Supplementary Table 6. Histological parameters measured in the anterior intestine of gilthead seabream fed the control and two basal diets supplemented with a mixture of pungent spices (capsicum, black pepper, ginger and cinnamaldehyde) at a dietary inclusion level of 0.1% ($SPICY_{0.1\%}$) and 0.15% ($SPICY_{0.15\%}$).

	**Control**	**$SPICY_{0.1\%}$**	**$SPICY_{0.15\%}$**
Villus height (µm)	762.66 ± 97.69	700.67 ± 134.84	704.37 ± 170.17
Height of enterocytes (µm)	33.28 ± 3.75	32.19 ± 3.59	29.83 ± 3.77
Density of goblet cells (nº cels/100 µm)	4.03 ± 0.42	4.28 ± 0.86	4.38 ± 1.32
Musculature thickness (µm)	87.42 ± 19.80	100.08 ± 29.93	86.62 ± 25.63

Values are represented as mean ± SD (n = 4 tanks per dietary group).

Supplementary Table 7. Values of the alpha diversity from the microbial communities of the anterior and posterior intestine from gilthead seabream fed the control and a basal diet supplemented with a mixture of pungent spices (capsicum, black pepper, ginger and cinnamaldehyde) at a dietary inclusion level of 0.1% ($SPICY_{0.1\%}$).

	**Anterior intestine**		**Posterior intestine**	
	Control	$SPICY_{0.1\%}$	Control	$SPICY_{0.1\%}$
ACE	573.27 ± 55.45	743.45 ± 139.96	491.86 ± 59.29	500.24 ± 64.04
Shannon	5.66 ± 0.16	5.75 ± 0.16	5.15 ± 0.31	5.64 ± 0.13
Simpson	0.98 ± 0.01	0.99 ± 0.00	0.96 ± 0.02^{a}	0.99 ± 0.00^{b}
Faith's PD	96.05 ± 9.38	90.29 ± 7.56	63.52 ± 8.53	70.19 ± 6.64

Values are represented as mean ± SEM (n = 12 fish per dietary group) and differences among groups ($P \leq 0.05$) are indicated by the different superscript letters.

Supplementary Table 8. Relative abundances of the dominant bacterial phyla (≥1.0%) from the microbial communities of the anterior and posterior intestine in gilthead seabream fed the control and two basal diets supplemented with a mixture of pungent spices (capsicum, black pepper, ginger and cinnamaldehyde) at a dietary inclusion level of 0.1% ($SPICY_{0.1\%}$).

	**Anterior intestine**			**Posterior intestine**		
	Control	$SPICY_{0.1\%}$	*P*	Control	$SPICY_{0.1\%}$	*P*
Firmicutes	31.16 ± 3.09	32.55 ± 4.36	0.367	36.16 ± 2.65	36.05 ± 2.74	> 0.999
Proteobacteria	29.35 ± 3.89	26.76 ± 3.03	0.348	30.90 ± 5.95	22.98 ± 2.73	0.574
Bacteroidota	19.21 ± 1.50	21.08 ± 1.58	0.303	17.11 ± 2.27	20.22 ± 1.77	0.604
Actinobacteriota	4.77 ± 0.42	4.62 ± 0.43	0.367	4.98 ± 0.56	4.62 ± 0.33	> 0.999
Unassigned	4.06 ± 0.61	3.65 ± 0.42	0.320	1.75 ± 0.36	3.60 ± 0.56	**0.030**
Campylobacterota	1.60 ± 0.28	2.94 ± 1.39	0.303	2.21 ± 0.38	1.87 ± 0.38	> 0.999
Desulfobacterota	1.88 ± 0.41	1.57 ± 0.28	0.340	2.22 ± 0.68	2.60 ± 0.39	> 0.999
Verrucomicrobiota	1.89 ± 0.41	1.64 ± 0.37	0.340	1.06 ± 0.20	1.30 ± 0.28	0.905
Chloroflexi	1.46 ± 0.60	0.98 ± 0.26	0.303	0.43 ± 0.07	1.31 ± 0.33	**0.028**

Values are represented as mean ± SEM (n = 12 fish per dietary group) and differences among groups ($P \leq 0.05$) are indicated in bold.

Supplementary Table 9. Relative abundances of the dominant bacterial genera (≥1.0%) from the microbial communities of the anterior and posterior intestine in gilthead seabream fed the control and two basal diets supplemented with a mixture of pungent spices (capsicum, black pepper, ginger and cinnamaldehyde) at a dietary inclusion level of 0.1% ($SPICY_{0.1\%}$).

	**Anterior intestine**			**Posterior intestine**		
	Control	$SPICY_{0.1\%}$	*P*	Control	$SPICY_{0.1\%}$	*P*
Firmicutes|Clostridia|Lachnospirales|Lachnospiraceae|Unassigned	4.48 ± 0.85	5.07 ± 1.19	0.238	4.03 ± 0.73	6.37 ± 1.14	**0.024**
Unassigned|Unassigned|Unassigned|Unassigned|Unassigned	4.06 ± 0.61	3.65 ± 0.42	0.207	1.75 ± 0.36	3.60 ± 0.56	**< 0.001**
Firmicutes|Clostridia|Peptostreptococcales-Tissierellales|Family XI|*Fenollaria*	2.69 ± 0.63	2.79 ± 0.74	0.286	3.78 ± 0.89	2.47 ± 0.76	0.084
Bacteroidota|Bacteroidia|Bacteroidales|Muribaculaceae|Unassigned	2.49 ± 0.42	2.60 ± 0.61	0.238	2.58 ± 0.56	3.35 ± 0.54	0.092
Proteobacteria|Gammaproteobacteria|Pseudomonadales|Pseudomonadaceae|*Pseudomonas*	2.64 ± 0.53	2.36 ± 0.65	0.243	2.85 ± 0.79	2.67 ± 0.76	0.162
Bacteroidota|Bacteroidia|Bacteroidales|Bacteroidaceae|*Bacteroides*	2.13 ± 0.38	2.18 ± 0.58	0.297	2.70 ± 0.49	3.44 ± 0.53	0.079
Bacteroidota|Bacteroidia|Bacteroidales|Porphyromonadaceae|*Porphyromonas*	2.02 ± 0.45	2.06 ± 0.53	0.280	2.88 ± 0.61	1.77 ± 0.51	0.057
Proteobacteria|Gammaproteobacteria|Pseudomonadales|Moraxellaceae|*Acinetobacter*	1.62 ± 0.36	1.67 ± 0.46	0.270	2.55 ± 0.76	1.96 ± 0.65	0.112
Firmicutes|Clostridia|Peptostreptococcales-Tissierellales|Family XI|*Ezakiella*	2.02 ± 0.50	1.73 ± 0.48	0.230	2.21 ± 0.57	1.52 ± 0.47	0.091
Firmicutes|Bacilli|Lactobacillales|Streptococcaceae|*Streptococcus*	1.45 ± 0.31	2.77 ± 1.85	0.275	1.19 ± 0.23	0.83 ± 0.22	0.078
Proteobacteria|Gammaproteobacteria|Enterobacterales|Vibrionaceae|*Catenococcus*	0.19 ± 0.19	0.01 ± 0.01	0.248	5.90 ± 4.65	0.18 ± 0.11	0.070
Bacteroidota|Bacteroidia|Flavobacteriales|Weeksellaceae|Unassigned	1.51 ± 0.36	1.08 ± 0.27	0.153	1.56 ± 0.35	1.79 ± 0.57	0.140
Campylobacterota|Campylobacteria|Campylobacterales|Campylobacteraceae|*Campylobacter*	1.29 ± 0.30	1.28 ± 0.38	0.296	1.81 ± 0.42	1.03 ± 0.31	**0.048**
Actinobacteriota|Actinobacteria|Corynebacteriales|Corynebacteriaceae|*Corynebacterium*	1.18 ± 0.27	1.23 ± 0.35	0.288	1.71 ± 0.28	1.07 ± 0.30	**0.039**
Desulfobacterota|Desulfovibrionia|Desulfovibrionales|Desulfovibrionaceae|*Desulfovibrio*	1.21 ± 0.28	1.03 ± 0.22	0.239	1.34 ± 0.40	1.62 ± 0.36	0.133
Proteobacteria|Alphaproteobacteria|Rhodobacterales|Rhodobacteraceae|*Marivita*	3.66 ± 3.11	0.48 ± 0.20	0.188	0.17 ± 0.11	0.08 ± 0.05	0.129
Firmicutes|Clostridia|Peptostreptococcales-Tissierellales|Family XI|*Peptoniphilus*	0.92 ± 0.20	0.91 ± 0.22	0.293	1.60 ± 0.31	0.94 ± 0.30	**0.039**
Bacteroidota|Bacteroidia|Bacteroidales|Prevotellaceae|*Prevotella*	1.06 ± 0.15	1.13 ± 0.25	0.254	1.25 ± 0.18	0.81 ± 0.23	0.051
Firmicutes|Clostridia|Peptostreptococcales-Tissierellales|Family XI|*Anaerococcus*	0.80 ± 0.22	1.02 ± 0.23	0.197	1.49 ± 0.33	0.85 ± 0.33	0.057
Proteobacteria|Alphaproteobacteria|Sphingomonadales|Sphingomonadaceae|*Sphingomonas*	1.09 ± 0.27	0.99 ± 0.22	0.268	1.26 ± 0.26	0.79 ± 0.23	0.057
Proteobacteria|Gammaproteobacteria|Burkholderiales|Burkholderiaceae|*Ralstonia*	0.83 ± 0.12	0.83 ± 0.18	0.282	1.23 ± 0.23	1.19 ± 0.25	0.157

Values are represented as mean ± SEM (n = 12 fish per dietary group) and differences among groups ($P \leq 0.05$) are indicated in bold.

Supplementary Table 10. Relative gene expression of liver mRNA transcripts of 48 h fasted- and 2 h postprandial- gilthead seabream fed the control and the basal diet supplemented with a mixture of pungent spices (capsicum, black pepper, ginger and cinnamaldehyde) at a dietary inclusion level of 0.1% (SPICY$_{0.1\%}$). All data are in reference to the expression levels of *grp-170* in 2 h postprandial-fish fed the control diet with an arbitrary value of 1.

	**Fed (Student's t-test)**			**Fasted (Student's t-test)**			**Two-way ANOVA**		
	Control	SPICY$_{0.1\%}$	P-value	Control	SPICY$_{0.1\%}$	P-value	Diet	Feeding time	Diet x Feeding time
fasn	1.84 ± 0.56	6.27 ± 0.89	**0.002**	0.21 ± 0.09	0.52 ± 0.31	0.331	**<0.001**	**<0.001**	**0.002**
elovl1	7.20 ± 1.24	7.16 ± 0.62	0.982	7.77 ± 0.61	8.38 ± 0.84	0.566	0.741	0.311	0.713
elovl4	0.33 ± 0.04	0.38 ± 0.04	0.365	0.30 ± 0.04	0.34 ± 0.05	0.482	0.252	0.466	0.903
elovl5	5.86 ± 1.35	4.65 ± 0.65	0.436	2.99 ± 0.96	1.97 ± 0.69	0.416	0.263	**0.008**	0.925
elovl6	10.04 ± 2.40	17.95 ± 2.53	**0.040**	3.44 ± 0.96	3.98 ± 1.12	0.719	**0.044**	**<0.001**	**0.076**
fads2	10.63 ± 2.09	11.91 ± 1.65	0.638	6.21 ± 0.91	8.09 ± 1.44	0.289	0.342	**0.018**	0.854
scd1a	6.65 ± 1.59	9.31 ± 1.40	0.230	1.22 ± 0.25	3.03 ± 1.22	0.174	**0.092**	**<0.001**	0.743
scd1b	27.20 ± 7.20	44.46 ± 4.51	**0.062**	2.83 ± 1.81	9.67 ± 4.71	0.178	**0.029**	**<0.001**	0.327
cyp7a1	0.67 ± 0.15	1.26 ± 0.14	**0.014**	1.59 ± 0.35	1.56 ± 0.24	0.943	0.254	**0.015**	0.205
pltp	0.13 ± 0.03	0.10 ± 0.02	0.342	0.16 ± 0.02	0.18 ± 0.02	0.695	0.584	**0.023**	0.312
atgl	0.03 ± 0.01	0.02 ± 0.00	0.441	0.37 ± 0.09	0.39 ± 0.13	0.915	0.949	**<0.001**	0.887
hl	4.71 ± 0.64	3.55 ± 0.28	0.118	6.64 ± 0.66	6.38 ± 0.96	0.823	0.290	**0.001**	0.501
lpl	2.11 ± 0.34	1.04 ± 0.16	**0.012**	6.64 ± 1.26	4.15 ± 0.88	0.138	**0.037**	**<0.001**	0.387
pla2g6	0.34 ± 0.04	0.37 ± 0.05	0.730	0.30 ± 0.06	0.39 ± 0.05	0.306	0.310	0.838	0.572
hnf4a	0.69 ± 0.09	0.81 ± 0.10	0.400	1.62 ± 0.10	1.57 ± 0.19	0.815	0.807	**<0.001**	0.524
srebp1	1.74 ± 0.36	2.41 ± 0.34	0.202	0.91 ± 0.11	1.95 ± 0.45	**0.040**	**0.018**	**0.069**	0.583
srebp2	0.26 ± 0.04	0.34 ± 0.04	0.210	0.34 ± 0.05	0.41 ± 0.08	0.469	0.208	0.247	0.941
fxr	0.35 ± 0.06	0.24 ± 0.02	0.109	0.64 ± 0.02	0.71 ± 0.12	0.540	0.841	**<0.001**	0.195
lxrα	0.51 ± 0.07	0.56 ± 0.05	0.576	1.07 ± 0.07	0.95 ± 0.08	0.251	0.558	**<0.001**	0.209
pparα	0.80 ± 0.16	0.64 ± 0.09	0.427	2.63 ± 0.36	2.00 ± 0.38	0.248	0.168	**<0.001**	0.403
pparβ	0.36 ± 0.08	0.20 ± 0.02	**0.078**	0.86 ± 0.13	0.70 ± 0.15	0.402	0.132	**<0.001**	0.973
pparγ	0.60 ± 0.05	0.74 ± 0.07	0.125	0.72 ± 0.06	0.87 ± 0.09	0.211	**0.051**	**0.100**	0.993
cpt1a	0.27 ± 0.05	0.25 ± 0.03	0.603	0.59 ± 0.08	0.47 ± 0.07	0.306	0.255	**<0.001**	0.491
hadh	0.99 ± 0.08	0.89 ± 0.08	0.395	2.44 ± 0.42	2.14 ± 0.51	0.655	0.568	**<0.001**	0.772
fat/cd36	0.00 ± 0.00	0.00 ± 0.00	0.619	0.02 ± 0.00	0.02 ± 0.01	0.762	0.661	**0.002**	0.884
h-fabp	44.93 ± 4.82	46.16 ± 2.89	0.831	62.10 ± 6.72	61.60 ± 6.72	0.959	0.948	**0.006**	0.877
cs	0.84 ± 0.10	1.02 ± 0.14	0.316	0.68 ± 0.04	0.96 ± 0.17	0.136	**0.074**	0.381	0.695
nd2	21.56 ± 2.61	24.02 ± 3.18	0.559	30.51 ± 3.94	35.91 ± 3.12	0.301	0.236	**0.003**	0.655
nd5	6.33 ± 0.62	7.36 ± 1.02	0.402	8.82 ± 0.84	8.36 ± 0.82	0.697	0.742	**0.047**	0.380
coxi	12.96 ± 1.51	14.13 ± 2.63	0.706	23.88 ± 2.93	21.68 ± 2.69	0.591	0.839	**<0.001**	0.507
pgc1α	0.02 ± 0.00	0.01 ± 0.00	0.233	0.06 ± 0.01	0.06 ± 0.01	0.875	0.877	**<0.001**	0.644
sirt1	0.08 ± 0.01	0.07 ± 0.01	0.523	0.12 ± 0.01	0.12 ± 0.01	1.000	0.725	**<0.001**	0.725
sirt2	0.11 ± 0.02	0.10 ± 0.01	0.436	0.14 ± 0.01	0.14 ± 0.02	0.914	0.693	**0.009**	0.569
cat	20.33 ± 2.74	22.42 ± 2.08	0.554	18.72 ± 1.40	19.99 ± 2.45	0.659	0.456	0.371	0.856
ucp1	9.44 ± 2.15	7.99 ± 1.48	0.589	17.47 ± 1.56	14.41 ± 2.87	0.365	0.290	**0.002**	0.701
gpx1	0.89 ± 0.10	0.86 ± 0.08	0.829	1.66 ± 0.20	1.64 ± 0.23	0.933	0.871	**<0.001**	0.994
gpx4	9.75 ± 2.14	8.78 ± 0.44	0.660	8.89 ± 0.98	9.12 ± 1.09	0.879	0.781	0.847	0.654
prdx3	0.84 ± 0.10	0.81 ± 0.08	0.793	0.78 ± 0.07	0.91 ± 0.07	0.217	0.570	0.858	0.326
prdx5	0.86 ± 0.07	0.78 ± 0.07	0.425	0.75 ± 0.07	1.05 ± 0.11	**0.034**	0.172	0.342	**0.025**
cu-zn-sod / sod1	4.11 ± 0.54	4.16 ± 0.39	0.940	4.87 ± 0.26	5.17 ± 0.42	0.548	0.669	**0.039**	0.760
mn-sod / sod2	1.25 ± 0.23	1.34 ± 0.10	0.726	1.05 ± 0.11	1.36 ± 0.18	0.160	0.228	0.581	0.498
grp-170	1.02 ± 0.06	1.14 ± 0.10	0.299	1.38 ± 0.31	1.55 ± 0.29	0.699	0.513	**0.090**	0.922
grp-94	2.31 ± 0.27	2.86 ± 0.45	0.311	3.76 ± 0.98	4.07 ± 1.36	0.855	0.628	0.141	0.894
grp-75	2.12 ± 0.26	2.38 ± 0.30	0.520	0.74 ± 0.07	1.12 ± 0.24	0.133	0.181	**<0.001**	0.821

Values are represented as mean ± SD (n = 8 fish per dietary group). Significant differences ($P \leq 0.05$) are marked in dark bold, and tendencies ($P \leq 0.1$) in red bold.

Supplementary Table 11. Relative gene expression of intestinal mRNA transcripts of 48 h fasted- and 2 h postprandial- gilthead seabream fed the control and the basal diet supplemented with a mixture of pungent spices (capsicum, black pepper, ginger and cinnamaldehyde) at a dietary inclusion level of 0.1% ($SPICY_{0.1\%}$). All data are in reference to the expression levels of *hes1-b* in 2 h postprandial-fish fed the control diet with an arbitrary value of 1.

	**Fed (Student's t-test)**			**Fasted (Student's t-test)**			**Two-way ANOVA**		
	Control	$SPICY_{0.1\%}$	P-value	Control	$SPICY_{0.1\%}$	P-value	Diet	Feeding time	Diet x Feeding time
pcna	7.41 ± 0.39	6.69 ± 0.45	0.241	3.18 ± 0.50	4.51 ± 0.55	**0.097**	0.525	**<0.001**	**0.038**
hes1-b	1.03 ± 0.10	0.88 ± 0.08	0.274	0.99 ± 0.15	0.93 ± 0.14	0.762	0.378	0.990	0.713
klf4	2.63 ± 0.46	2.70 ± 0.36	0.918	1.32 ± 0.23	1.21 ± 0.21	0.746	0.949	**<0.001**	0.805
cldn12	0.40 ± 0.03	0.41 ± 0.03	0.733	0.42 ± 0.04	0.41 ± 0.02	0.790	0.966	0.762	0.666
cldn15	13.73 ± 0.77	13.83 ± 0.89	0.934	19.19 ± 1.26	20.10 ± 1.34	0.627	0.646	**<0.001**	0.711
cdh1	6.55 ± 0.46	6.59 ± 0.46	0.944	6.12 ± 0.42	6.38 ± 0.40	0.665	0.731	0.471	0.813
cdh17	31.29 ± 1.61	29.80 ± 1.70	0.535	33.14 ± 4.26	33.44 ± 3.61	0.958	0.846	0.372	0.769
tjp1	0.39 ± 0.02	0.38 ± 0.03	0.770	0.43 ± 0.10	0.30 ± 0.03	0.223	0.203	0.718	0.274
dsp	2.89 ± 0.24	3.26 ± 0.23	0.284	3.63 ± 0.33	3.19 ± 0.17	0.245	0.879	0.190	0.111
cx32.2	20.12 ± 2.65	17.86 ± 2.59	0.552	42.70 ± 6.68	44.90 ± 5.25	0.800	0.995	**<0.001**	0.634
cxadr	1.51 ± 0.06	1.67 ± 0.07	0.119	2.31 ± 0.14	1.99 ± 0.09	**0.070**	0.405	**<0.001**	**0.017**
alpi	21.76 ± 1.91	16.77 ± 2.54	0.139	41.77 ± 4.97	46.62 ± 4.65	0.488	0.986	**<0.001**	0.192
fabp1	38.22 ± 1.59	40.54 ± 4.70	0.648	51.91 ± 4.89	35.67 ± 5.46	**0.044**	0.127	0.327	**0.045**
fabp2	207.10 ± 44.47	259.00 ± 57.41	0.487	443.90 ± 68.08	267.55 ± 34.38	**0.037**	0.247	**0.027**	**0.039**
fabp6	0.12 ± 0.02	0.22 ± 0.09	0.276	0.02 ± 0.00	0.02 ± 0.01	0.909	0.253	**0.001**	0.244
muc2	16.64 ± 1.42	17.43 ± 1.51	0.708	21.45 ± 3.57	19.35 ± 1.20	0.585	0.763	0.128	0.506
muc13	28.04 ± 1.77	30.29 ± 2.97	0.541	35.77 ± 4.23	32.54 ± 1.78	0.492	0.869	0.101	0.359
tnf-α	0.20 ± 0.03	0.17 ± 0.02	0.441	0.14 ± 0.01	0.13 ± 0.01	0.563	0.337	**0.015**	0.665
il-1β	0.09 ± 0.01	0.12 ± 0.01	**0.080**	0.06 ± 0.00	0.07 ± 0.01	0.479	**0.060**	**<0.001**	0.272
il-6	0.14 ± 0.02	0.14 ± 0.03	0.944	0.05 ± 0.01	0.04 ± 0.01	0.334	0.756	**<0.001**	0.863
il-7	0.47 ± 0.05	0.40 ± 0.04	0.295	0.31 ± 0.03	0.32 ± 0.02	0.750	0.442	**0.005**	0.279
il-8	0.14 ± 0.01	0.17 ± 0.03	0.204	0.18 ± 0.03	0.16 ± 0.02	0.668	0.691	0.639	0.271
il-10	0.15 ± 0.02	0.15 ± 0.03	1.000	0.08 ± 0.01	0.07 ± 0.01	0.213	0.697	**<0.001**	0.697
il-12β	0.35 ± 0.03	0.43 ± 0.06	0.263	0.28 ± 0.04	0.24 ± 0.03	0.397	0.719	**0.004**	0.160
il-15	0.19 ± 0.01	0.18 ± 0.01	0.701	0.33 ± 0.04	0.25 ± 0.02	**0.093**	**0.078**	**<0.001**	0.128
il-34	0.56 ± 0.04	0.51 ± 0.03	0.283	1.05 ± 0.06	0.81 ± 0.06	**0.010**	**0.005**	**<0.001**	**0.055**
cd4-1	0.13 ± 0.01	0.14 ± 0.02	0.333	0.23 ± 0.02	0.17 ± 0.02	**0.040**	0.272	**<0.001**	**0.024**
cd8b	0.04 ± 0.01	0.04 ± 0.01	0.554	0.06 ± 0.01	0.03 ± 0.00	**0.007**	**0.011**	0.805	**0.067**
ccr3	0.36 ± 0.02	0.38 ± 0.04	0.607	0.55 ± 0.05	0.50 ± 0.02	0.423	0.748	**<0.001**	0.338
ccr9	0.51 ± 0.04	0.65 ± 0.06	**0.072**	1.60 ± 0.20	1.26 ± 0.11	0.147	0.369	**<0.001**	**0.044**
ccr11	1.45 ± 0.12	1.64 ± 0.18	0.419	3.09 ± 0.26	2.82 ± 0.23	0.455	0.858	**<0.001**	0.288
ck8 / ccl20	1.71 ± 0.22	2.40 ± 0.33	0.119	3.80 ± 0.52	3.00 ± 0.49	0.291	0.892	**0.003**	**0.087**
csf1r1	0.41 ± 0.04	0.41 ± 0.04	1.000	0.48 ± 0.06	0.45 ± 0.02	0.578	0.693	0.252	0.693
igm	4.68 ± 0.99	4.81 ± 0.61	0.918	7.31 ± 1.17	8.50 ± 2.83	0.719	0.714	**0.086**	0.765
igt	0.27 ± 0.04	0.35 ± 0.04	0.164	0.30 ± 0.03	0.28 ± 0.03	0.709	0.370	0.540	0.177
lgals1	6.20 ± 0.30	6.66 ± 0.40	0.374	7.92 ± 0.67	7.04 ± 0.54	0.318	0.671	**0.043**	0.186
lgals8	2.15 ± 0.16	2.02 ± 0.16	0.579	2.07 ± 0.22	2.22 ± 0.11	0.532	0.938	0.714	0.399
tlr2	0.28 ± 0.04	0.25 ± 0.03	0.512	0.44 ± 0.03	0.38 ± 0.02	0.136	0.134	**<0.001**	0.601
tlr5	0.10 ± 0.01	0.09 ± 0.01	0.702	0.04 ± 0.00	0.03 ± 0.00	0.193	0.429	**<0.001**	0.951
tlr9	0.13 ± 0.02	0.12 ± 0.02	0.761	0.05 ± 0.01	0.04 ± 0.01	0.348	0.560	**<0.001**	1.000
cd209d	0.14 ± 0.01	0.17 ± 0.02	0.176	0.19 ± 0.01	0.17 ± 0.02	0.401	0.643	0.149	0.112
cd302	3.68 ± 0.18	3.97 ± 0.16	0.254	5.93 ± 0.28	5.04 ± 0.30	**0.049**	0.215	**<0.001**	**0.020**
mrc1	0.59 ± 0.04	0.60 ± 0.06	0.868	0.96 ± 0.11	0.78 ± 0.05	0.170	0.240	**<0.001**	0.185
fcl	0.62 ± 0.15	0.73 ± 0.20	0.661	3.77 ± 0.81	3.21 ± 1.13	0.701	0.750	**<0.001**	0.637

Values are represented as mean ± SD (n = 8 fish per dietary group). Significant differences ($P \leq 0.05$) are marked in dark bold, and tendencies ($P \leq 0.1$) in red bold.

Supplementary Table 12. Extended summary of referenced studies compiling the individual effect of the dietary supplementation / incorporation of capsicum, black pepper, ginger and cinnamaldehyde on the growth and feed performances of several fish species.

Spice / Active principle	Fish species	Period of feeding	Remarkable particularities during the feeding trial	Diet composition (dry-matter basis)	Additive composition (and origin, if provided)	Additive format	Dietary inclusion levels (%)	Growth performance	Feed performance	Reference
Capsicum (*Capsicum annuum*)	Gilthead seabream (*Sparus aurata*)	6 weeks (6 days a week)	N/I	48% CP + 13% CF	Edible portion of capsicum devoid of stem ends, seeds, and core (purchased from a local market on Alexandria, harvested on 2007 season)	Meal / powder	0.3	= BW<br>= WG<br>= SGR	= FI<br>= FCR	Wassef et al. (2010)
	Blue streak hap (*Labidochromis caeruleus*)	45 days	N/I	34% CP + 13% CF	Capsicum	N/I	2	= BW<br>= WGR<br>= SGR	= FCR	Yılmaz and Ergün (2011)
							5	= BW<br>= WGR<br>= SGR	= FCR	
	Mozambique tilapia (*Oreochromis mossambicus*)	45 days	N/I	37% CP + 10% CF	Capsicum	Oleoresin (purchased from Kutluer, Turkey)	0.7	= BW<br>= WG<br>= SGR	= FCR	Yılmaz et al. (2013)
							1.4	= BW<br>= WG<br>= SGR	= FCR	
	Jewel cichlid (*Hemichromis guttatus*)	90 days	N/I	37% CP + 11% CF	Capsicum (purchased from a local market in Trukey)	Flour	3	= BW<br>= WG<br>= SGR	= FCR	Yigit et al. (2021)
							7	= BW<br>= WG<br>= SGR	= FCR	
							11	= BW<br>= WG<br>= SGR	= FCR	
							15	= BW<br>= WG<br>= SGR	= FCR	

Spice / Active principle	Fish species	Period of feeding	Remarkable particularities during the feeding trial	Diet composition (dry-matter basis)	Additive composition (and origin, if provided)	Additive format	Dietary inclusion levels (%)	Growth performance	Feed performance	Reference
Capsicum (*Capsicum annuum*)	Rainbow trout (*Onchorhynchus mykiss*)	20 days	N/I	45% CP + 20% CF	Leaves of capsicum (Kahramanmaraş, Turkey, harvested in September)	Meal (extracted with acetone)	0.5	= BW	N/I	Yanar et al. (2016)
							2	= BW	N/I	
							4.4	= BW	N/I	
		40 days					0.5	= BW	N/I	
							2	= BW	N/I	
							4.4	= BW	N/I	
		60 days					0.5	= BW	N/I	
							2	= BW	N/I	
							4.4	= BW	N/I	
		80 days					0.5	= BW<br>= TL<br>= SGR	= FCR	
							2	= BW<br>= TL<br>= SGR	= FCR	
							4.4	= BW<br>= TL<br>= SGR	= FCR	
		20 days	N/I	40% CP + 12% CF	Capsicum (provided by local producers from Iran)	Powder	0.0033	= BW<br>↑ TL	N/I	Talebi et al. (2013)
							0.0044	↑ BW<br>↑ TL	N/I	
							0.0055	↑ BW<br>↑ TL	N/I	
		40 days					0.0033	↑ BW<br>↑ TL	N/I	
							0.0044	↑ BW<br>↑ TL	N/I	
							0.0055	↑ BW<br>↑ TL	N/I	
		60 days					0.0033	↑ BW<br>↑ TL<br>= SGR<br>= WGR	= FCR	
							0.0044	↑ BW<br>↑ TL<br>= WGR<br>= SGR	= FCR	
							0.0055	↑ BW<br>↑ TL<br>= WGR<br>= SGR	= FCR	

Spice / Active principle	Fish species	Period of feeding	Remarkable particularities during the feeding trial	Diet composition (dry-matter basis)	Additive composition (and origin, if provided)	Additive format	Dietary inclusion levels (%)	Growth performance	Feed performance	Reference
Black pepper (*Piper nigrum*) / piperine	African catfish *(Clarias gariepinus)*	30 days	N/I	31% CP + 7% CF supplemented with turmeric at 0.5%	Black pepper (purchased from a local market in Zagazig city, Egypt)	Powder	0.1	= BW<br>= WG<br>= SGR	N/I	El-Houseiny et al. (2019)
			Fish were exposed to cadmium at 0.8 mg/L in the water					= BW<br>= WG<br>= SGR	N/I	
	Rainbow trout (*Oncorhynchus mykiss*)	30 days	N/I	45% CP + 16% CF	Black pepper	Powder (manufactured by P.I.C.Co LTD)	1	= BW	N/I	Stoev and Zhelyazkov (2021)
		60 days						= BW<br>= WG	↓ FCR	
	Common carp (*Cyprinus carpio*)	40 days	N/I	53% CP + 7% CF (a diet based on casein, gelatin and a fatty acid mixture with methionine at 0.4%)	Piperine	Powder (> 97.0; manufactured by Sigma-Aldrich)	0.02	= WG	↑ FCR	Wojno et al. (2021)
					Fruits of black pepper (purchased from a local vendor from Ohio)	Powder (extracted with hexane and filtered)	0.02	↓ WG	↑ FCR	
	Rohu fish (*Labeo rohita*)	12 weeks	N/I	34% CP (%CF N/I)	Leaves of black pepper (collected during the period from October and December 2019 from nursery farms in District Kasur, Pakistan)	Powder (extracted with methanol)	1	= TL<br>↓ FL<br>↑ WG	↑ FI<br>↑ FCR	Ullah et al. (2023)
							2	↑ TL<br>↑ FL<br>↑ WG	↑ FI<br>↑ FCR	
							3	↓ TL<br>↓ FL<br>= WG	↑ FI<br>↑ FCR	
Ginger (*Zingiber officinale)*	Asian sea bass (*Lates calcarifer*)	15 days	N/I	42% CP + 17% CF (wet-matter basis, 9.7% moisture)	Peeled rhizomes of ginger (purchased from the local market in Kuala Terengganu, Malaysia)	Powder	0.1	↑ WGR<br>↑ SGR	↓ FCR	Talpur et al. (2013)
							0.2	↑ WGR<br>↑ SGR	↓ FCR	
							0.3	↑ WGR<br>↑ SGR	↓ FCR	
							0.5	↑ WGR<br>↑ SGR	↓ FCR	
							1	↑ WGR<br>↑ SGR	↓ FCR	

Spice / Active principle	Fish species	Period of feeding	Remarkable particularities during the feeding trial	Diet composition (dry-matter basis)	Additive composition (and origin, if provided)	Additive format	Dietary inclusion levels (%)	Growth performance	Feed performance	Reference
Ginger (*Zingiber officinale)*	Nile tilapia (*Oreochromis niloticus*)	55 days	N/I	40% CP (%CF N/I; wet-matter basis, 9.7% moisture)	Rhizomes of ginger (harvested in the State of Amazonas, Brazil)	Essential oil (extracted by hydrodistillation method)	0.5	= BW<br>= TL<br>= SGR	= FCR	Brum et al. (2017)
							1	= BW<br>= TL<br>= SGR	= FCR	
							1.5	↓ BW<br>↓ TL<br>↓ SGR	↑ FCR	
	Rohu fish (*Labeo rohita*)	30 days	N/I	28% CP + 7% CF	Peeled rhizomes of ginger (purchased from a local market in Thanjavur, Tamil Nadu)	Powder	0.2	= WG<br>= SGR	= FI<br>= FCR	Sukumaran et al. (2016)
							0.4	= WG<br>= SGR	= FI<br>= FCR	
							0.6	= WG<br>= SGR	↑ FI<br>= FCR	
							0.8	↑ WG<br>↑ SGR	↑ FI<br>↓ FCR	
							1	= WG<br>↑ SGR	↑ FI<br>= FCR	
		60 days					0.2	= WG<br>= SGR	= FI<br>= FCR	
							0.4	= WG<br>= SGR	= FI<br>= FCR	
							0.6	↑ WG<br>↑ SGR	= FI<br>↓ FCR	
							0.8	↑ WG<br>↑ SGR	= FI<br>↓ FCR	
							1	↑ WG<br>↑ SGR	= FI<br>↓ FCR	
	Common carp (*Cyprinus carpio*)	60 days	Fish were reared in high stocking densities (20 kg/m^3)	41% CP + 9% CF	Ginger (purchased from a local shop in Iran)	Powder	0.5	= BW<br>↑ WGR<br>↑ SGR	↓ FCR	Fazelan et al. (2020)
							1	= BW<br>↑ WGR<br>↑ SGR	↓ FCR	

Spice / Active principle	Fish species	Period of feeding	Remarkable particularities during the feeding trial	Diet composition (dry-matter basis)	Additive composition (and origin, if provided)	Additive format	Dietary inclusion levels (%)	Growth performance	Feed performance	Reference
Ginger (*Zingiber officinale)*	Common carp (*Cyprinus carpio*)	60 days	N/I	38-41% CP + 4-16% CF (wet-matter basis, 5-11% moisture)	Rhizomes of ginger (obtained from Zarringiah medicinal plants company in Urmia, Iran)	Powder (extracted with ethanol)	0.1	= BW<br>= WG<br>↑ SGR	↓ FCR	Mohammadi et al. (2020)
							0.2	↑ BW<br>↑ WG<br>↑ SGR	↓ FCR	
							0.4	↑ BW<br>↑ WG<br>↑ SGR	↓ FCR	
Cinnamaldehyde	Nile tilapia (*Oreochromis niloticus*)	15 days	N/I	33% CP + 9% CF	Cinnamaldehyde (obtained from Flaka Chemical, Switzerland)	Essential oil (≥98%)	0.105	= BW<br>= WG	= FI<br>= FCR	Amer et al. (2018)
							0.210	= BW<br>= WG	= FI<br>= FCR	
		75 days					0.105	= BW<br>= WG<br>= SGR	= FI<br>= FCR	
							0.210	= BW<br>= WG<br>= SGR	= FI<br>= FCR	
		12 weeks	N/I	32% CP + 8% CF	Cinnamaldehyde (W228613), polyoxyethylene, monooleate (Tween 80, food grade), sodium alginate (medium viscosity, A-2033) (purchased from Sigma-Aldrich Co., St. Louis, USA)	Nanoemulsion (prepared with the above compounds)	0.01	↑ BW<br>↑ WG<br>↑ WGR<br>↑ SGR	↓ FI<br>↓ FCR	Abd El-Hamid et al. (2021)
							0.02	↑ BW<br>↑ WG<br>↑ WGR<br>↑ SGR	↓ FI<br>↓ FCR	
							0.03	↑ BW<br>↑ WG<br>↑ WGR<br>↑ SGR	↓ FI<br>↓ FCR	
	Grass carp (*Ctenopharyngodon idella*)	60 days	N/I	28% CP + 4% CF	Cinnamaldehyde (obtained from the Shanghai Menon Animal Nutrition Technology Co., Ltd., China) diluted to 18% with silicon dioxide to enhance it stability	Essential oil (>98%)	0.02 (0.0036 cinnamaldehyde)	↑ BW<br>↑ WGR<br>↑ SGR	↑ FI	Zhou et al. (2020)
							0.04 (0.0072 cinnamaldehyde)	↑ BW<br>↑ WGR<br>↑ SGR	↑ FI	
							0.06 (0.0108 cinnamaldehyde	↑ BW<br>↑ WGR<br>↑ SGR	↑ FI	
							0.08 (0.0144 cinnamaldehyde)	↑ BW<br>↑ WGR<br>↑ SGR	↑ FI	

Spice / Active principle	Fish species	Period of feeding	Remarkable particularities during the feeding trial	Diet composition (dry-matter basis)	Additive composition (and origin, if provided)	Additive format	Dietary inclusion levels (%)	Growth performance	Feed performance	Reference
Cinnamaldehyde	Tongue sole (*Cynoglossus semilaevis*)	60 days	N/I	52% CP + 14% CF	Cinnamaldehyde (N/I), lecithin, α-tocopherol, ethanol and potassium dihydrogen phosphate	Liposome-encapsulated product (prepared with the above compounds)	0.1	= BW<br>↑ WGR<br>↑ SGR	↓ FCR	Wang et al. (2021)
			Diet supplemented with 1 x 10^7 *Bacillus subtilis*					↑ BW<br>↑ WGR<br>↑ SGR	↓ FCR	
	Fat greenling (*Hexagrammos otakii*)	8 weeks	N/I	51% CP + 10% CF	Cinnamaldehyde (purchased from Improved McLin Biotech Co., Shanghai, China)	N/I	0.02	↑ WGR<br>↑ SGR	↓ FCR	Gu et al. (2022)
							0.04	↑ WGR<br>↑ SGR	↓ FCR	
							0.06	↑ WGR<br>↑ SGR	↓ FCR	
							0.08	↑ WGR<br>↑ SGR	↓ FCR	
							0.1	↑ WGR<br>↑ SGR	↓ FCR	

Abbreviations:

CP, crude protein
CF, crude fat
BW, body weight
TL, total length
FL, fork length
WG, weight gain (WG (g) = BW_f - BW_i)
WGR, Weight Gain Rate (WGR (%) = 100 x WG / BW_i)
SGR, specific growth rate
FI, feed intake
FCR, feed conversion ratio
N/I, not identified or not assessed

Supplementary Figure 1. Template rarefaction curves represented in function of observed ASVs from the anterior and posterior intestine of gilthead seabream (n = 12 per dietary group) fed the control and the basal diet supplemented with a mixture of mixture of pungent spices (capsicum, black pepper, ginger and cinnamaldehyde) at a dietary inclusion level of 0.1% ($SPICY_{0.1\%}$).

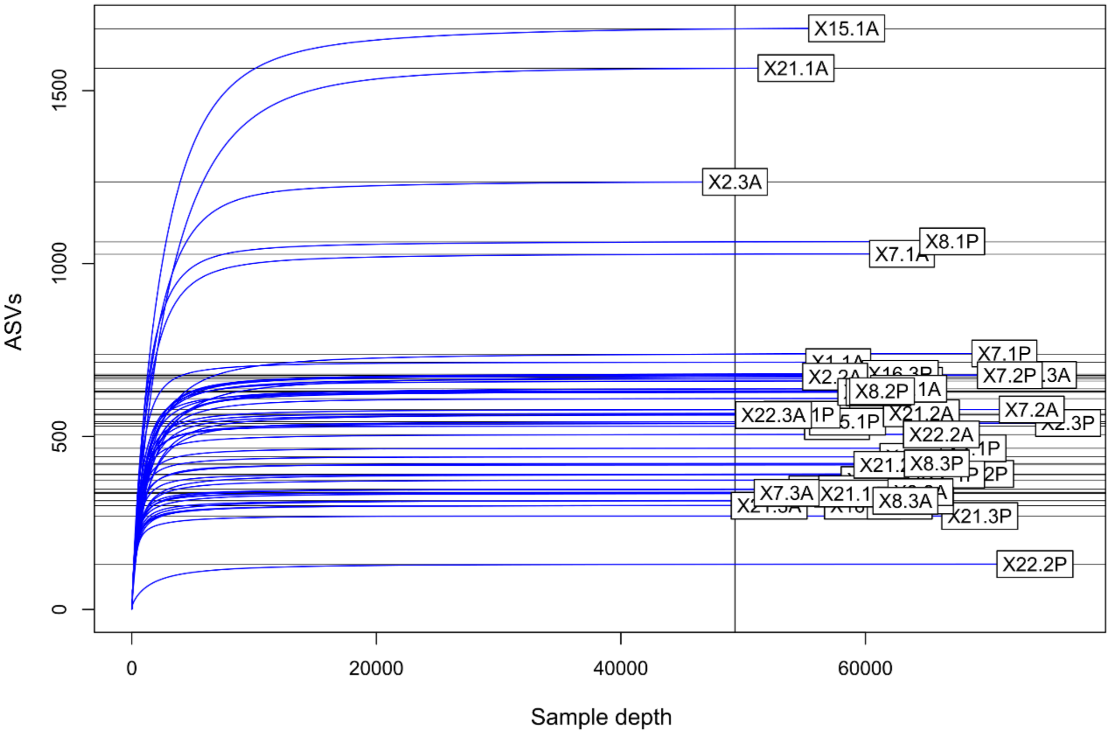

Supplementary Figure 2. Validation (permutation test) of the PLS-DA model constructed for analysing the separation among individuals based on gene expression tendencies ($P \leq 0.1$) of selected marker genes for liver in 48 h fasted- and 2 h postprandial-gilthead seabream (n = 8 per dietary group) fed the control and the basal diet supplemented with a mixture of pungent spices (capsicum, black pepper, ginger and cinnamaldehyde) at a dietary inclusion level of 0.1% (SPICY$_{0.1\%}$).

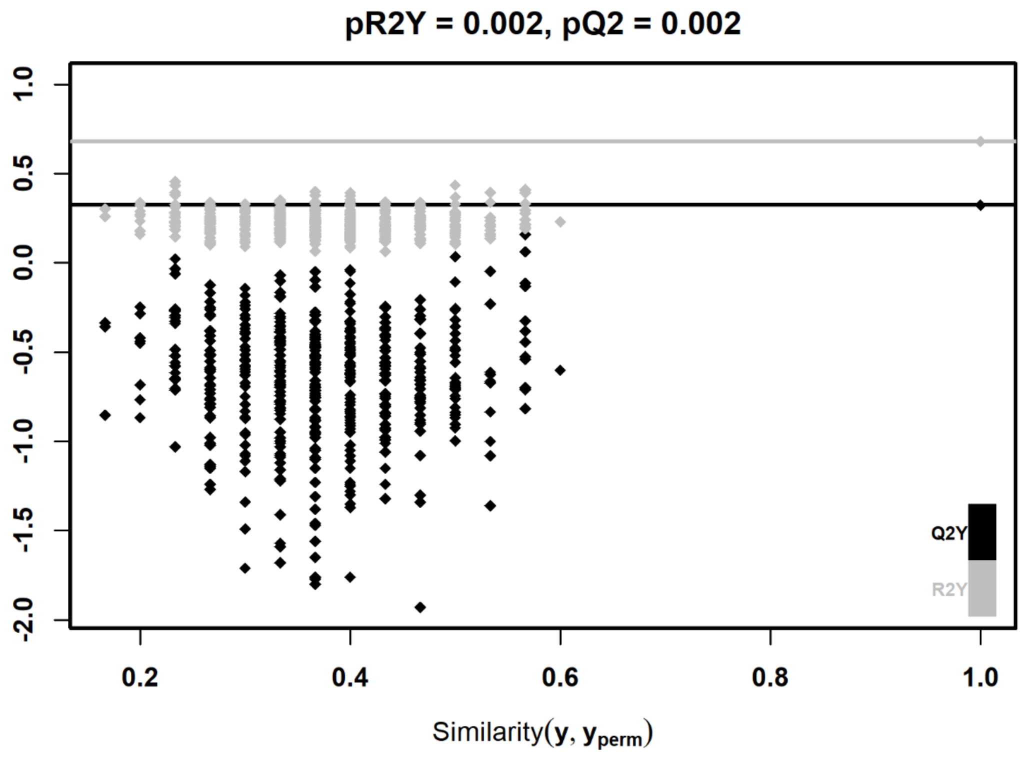

Supplementary Figure 3. Validation (permutation test) of the PLS-DA model constructed for analysing the separation among individuals based on gene expression tendencies ($P \leq 0.1$) of selected marker genes for anterior intestine in 48 h fasted- and 2 h postprandial- gilthead seabream (n = 8 per dietary group) fed the control and the basal diet supplemented with a mixture of pungent spices (capsicum, black pepper, ginger and cinnamaldehyde) at a dietary inclusion level of 0.1% ($SPICY_{0.1\%}$).

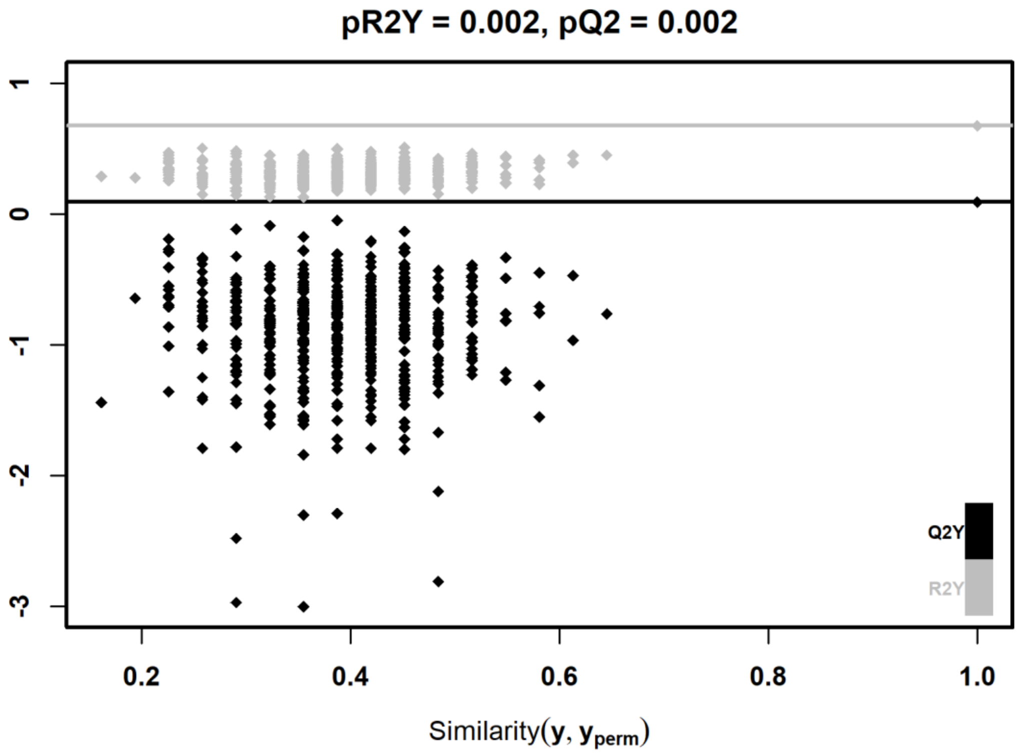

Supplement: Supplementary file 1 [file DataSheet_1.pdf]
